# Water enables mild oxidation of methane to methanol on gold single-atom catalysts

**DOI:** 10.1038/s41467-021-21482-z

**Published:** 2021-02-22

**Authors:** Laihao Luo, Jie Luo, Hongliang Li, Fangning Ren, Yifei Zhang, Andong Liu, Wei-Xue Li, Jie Zeng

**Affiliations:** Hefei National Laboratory for Physical Sciences at the Microscale, CAS Key Laboratory of Strongly-Coupled Quantum Matter Physics, Key Laboratory of Surface and Interface Chemistry and Energy Catalysis of Anhui Higher Education Institutes, Department of Chemical Physics, University of Science and Technology of China, Hefei, Anhui P. R. China

**Keywords:** Catalyst synthesis, Photocatalysis, Two-dimensional materials

## Abstract

As a 100% atom-economy process, direct oxidation of methane into methanol remains as a grand challenge due to the dilemma between activation of methane and over-oxidation of methanol. Here, we report that water enabled mild oxidation of methane into methanol with >99% selectivity over Au single atoms on black phosphorus (Au_1_/BP) nanosheets under light irradiation. The mass activity of Au_1_/BP nanosheets reached 113.5 μmol g_catal_^−1^ in water pressured with 33 bar of mixed gas (CH_4_:O_2_ = 10:1) at 90 °C under light irradiation (1.2 W), while the activation energy was 43.4 kJ mol^−1^. Mechanistic studies revealed that water assisted the activation of O_2_ to generate reactive hydroxyl groups and •OH radicals under light irradiation. Hydroxyl groups reacted with methane at Au single atoms to form water and CH_3_* species, followed by oxidation of CH_3_* via •OH radicals into methanol. Considering the recycling of water during the whole process, we can also regard water as a catalyst.

## Abstract (web summary)

It is important but challenging to oxidize methane by O_2_ into methanol under ambient conditions. Here, the authors achieved mild oxidation of methane into methanol over Au single atoms on black phosphorus nanosheets with the help of water under light irradiation.

## Introduction

Advances in hydraulic fracturing technology have enabled ongoing discovery of large reserves of natural gas, which primarily contains methane^1^. At present, most of produced methane is burned for heating, transport, and electricity-generation purposes. Meanwhile, methane is the second most relevant greenhouse gas emitted from anthropogenic activities, as global warming potential of methane is 25 times higher than that of CO_2_^2^. In this case, it is highly desired to develop efficient techniques for upgrading of methane. The current industrial route is via steam or dry reforming to generate syngas (a mixture of CO and H_2_), followed by Fischer–Tropsch synthesis or methanol synthesis^3,4^. Such route operated at high temperature (>700 °C) not only needs huge energy input, but also leads to the deactivation of catalysts because of coking^5^. Direct conversion of methane into chemicals or liquid fuels has been achieved under mild conditions^6–9^. For instance, aqueous Au-Pd colloids achieved selective oxidation of methane into methanol with H_2_O_2_ as an oxidant under 30 bar of CH_4_ at 50 °C^10^. Methane was reported to be oxidized into methanesulfonic acid by SO_3_ over an electrophilic initiator at 50 °C^11^. However, these processes generally involve corrosive oxidant or expensive media, such as H_2_O_2_, oleum, trifluoroacetic acid, and hydrobromic acid, which is not suitable for commercialization^12–15^.

Among direct processes, aerobic oxidation of methane into methanol is an ideal reaction with 100% atom economy (Eq. 1). The dilemma lies in activation of methane and over-oxidation of methanol^16–18^. Methane takes a non-polar tetrahedral structure with high dissociation energy of C–H bond (439.3 kJ mol^−1^), rendering great difficulties for activation^19–21^. As methane and O_2_ are in singlet and triplet ground states, respectively, the direct reaction of methane with O_2_ in ground states is spin-prohibition, which should be overcome^22^. Once methane is activated, the produced methanol tends to be over-oxidized into CO or CO_2_ from a thermodynamic perspective (Eqs. 1–3)^23^. Therefore, it is important but challenging to achieve highly selective oxidation of methane with O_2_ into methanol under ambient conditions.

1 $${\mathrm{CH}}_4 + 0.5{\mathrm{O}}_2 = {\mathrm{CH}}_3{\mathrm{OH}} \qquad \Delta {\mathrm{H}}_{298{\mathrm{K}}} = - 126.4{\mathrm{kJ}}\,{\mathrm{mol}}^{ - 1}$$

2 $${\mathrm{CH}}_4 + 1.5{\mathrm{O}}_2 = {\mathrm{CO}} + 2{\mathrm{H}}_2{\mathrm{O}}\qquad\Delta {\mathrm{H}}_{298{\mathrm{K}}} = - 519.6{\mathrm{kJ}}\,{\mathrm{mol}}^{ - 1}$$

3 $${\mathrm{CH}}_4 + 2{\mathrm{O}}_2 = {\mathrm{CO}}_2 + 2{\mathrm{H}}_2{\mathrm{O}}\qquad\Delta {\mathrm{H}}_{298{\mathrm{K}}} = - 802.6{\mathrm{kJmol}}^{ - 1}$$

Herein, we achieved mild oxidation of methane into methanol over Au single atoms on black phosphorus (Au_1_/BP) nanosheets with the help of water under light irradiation. Photocatalysis offers an efficient approach to drive thermodynamically non-spontaneous reactions under mild conditions^24,25^. We applied black phosphorus (BP) nanosheets as the support that affords broadband solar absorption for photocatalysis^26,27^. During methane oxidation, the mass activity of Au_1_/BP nanosheets was 113.5 μmol g_catal_^−1^ in water pressured with 33 bar of mixed gas (CH_4_:O_2_ = 10:1) at 90 °C under light irradiation (1.2 W). The selectivity for methanol reached as high as >99%. Based on mechanistic studies, water and O_2_ were activated on Au_1_/BP nanosheets to form reactive hydroxyl groups and •OH radicals under light irradiation. The reactive hydroxyl groups enabled mild oxidation of methane into CH_3_* species, followed by oxidation of CH_3_* via •OH radicals into methanol. As water is consumed to form hydroxyl groups and produced via reaction of hydroxyl groups with methane, water is completely recycled and thus can also be regarded as a catalyst.

## Results and discussion

### Synthesis and characterizations of Au_1_/BP nanosheets

**Fig. 1:**
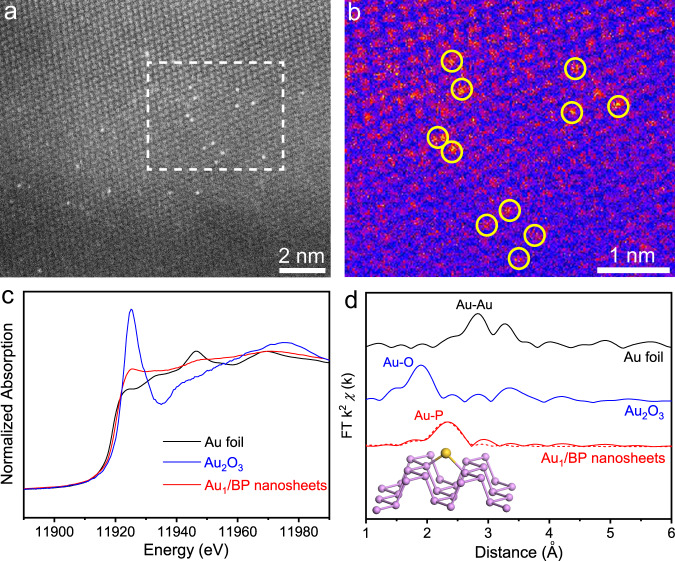
Structural characterizations of Au_1_/BP nanosheets. **a** HAADF-STEM image of Au_1_/BP nanosheets. **b** Magnified HAADF-STEM image with color-coded intensity. This image shows the region marked by dash box in **a**. **c** XANES spectrum and **d** EXAFS spectrum of Au_1_/BP nanosheets. Au foil and Au_2_O_3_ were used as the references. The inset image in **d** is a structural model of Au_1_/BP nanosheets. Violet and yellow spheres represent for P and Au atoms, respectively.

To prepare Au_1_/BP nanosheets, we first synthesized bulk BP through a low-pressure transport route according to the reported literature^28^. BP nanosheets were prepared by liquid exfoliation of bulk BP. The diameter of free-standing BP nanosheets was several hundred nanometers (Supplementary Fig. 1). As measured by atomic force microscopy, the thickness of the as-obtained nanosheet was ca. 3.0 nm (Supplementary Fig. 2). We prepared Au_1_/BP nanosheets by injecting HAuCl_4_ solution into a mixture containing ethanol and BP nanosheets with the use of a syringe pump under nitrogen protection. Figure 1a shows a high-angle annular dark-field scanning transmission electron microscopy (HAADF-STEM) image of Au_1_/BP nanosheets, where the Au mass loading was determined as 0.2 wt%. The magnified HAADF-STEM image and its corresponding color-coded intensity map were shown in Fig. 1b, indicating the isolated distribution of Au atoms in the absence of Au nanoparticles (NPs). This point was further verified by HAADF-STEM images with lower magnifications (Supplementary Fig. 3). By increasing the concentration of HAuCl_4_ solution, we obtained Au NPs on BP nanosheets (denoted as Au NPs/BP nanosheets). As shown in Supplementary Figure 4, Au NPs with an average size of 6 nm were uniformly dispersed on BP nanosheets with a mass loading of 1.0 wt%.

We further characterized the global structure of Au_1_/BP nanosheets. X-ray diffraction (XRD) patterns revealed that both BP and Au_1_/BP nanosheets were well indexed to the orthorhombic phase of BP (Supplementary Fig. 5). Especially, two intense diffraction peaks at 34.2° and 52.3° corresponded to (040) and (060) facets of orthorhombic BP, respectively, which are representative layered planes of BP. No patterns corresponding to Au phase were observed, in concert with the isolated dispersion of Au atoms. Raman spectra of BP and Au_1_/BP nanosheets show similar peaks located at 361, 438, and 466 cm^−1^, which were assigned to *A*_g_^1^(out-of-plane), *B*_2g_, (in-plane), and *A*_g_^2^(in-plane) vibration modes of BP, respectively (Supplementary Fig. 6).

To further determine the local environment in Au_1_/BP nanosheets, we measured X-ray absorption near-edge spectroscopy (XANES) and extended X-ray absorption fine structure (EXAFS). The Au *L*_3_-edge XANES profiles indicated that the Au species in Au_1_/BP nanosheets were in a mediate oxidation state (Fig. 1c), according to the mediate intensity for white line compared with Au foil and Au_2_O_3_. As shown in EXAFS in *R* space (Fig. 1d), the spectrum of Au_1_/BP nanosheets exhibited a prominent peak at 2.33 Å from the Au–P shell with a coordination number (*CN*) of 2.0 (Supplementary Table 1). No other typical peaks for Au–Au contribution at longer distances (>2.5 Å) or for Au–O contributions at shorter distance (<1.9 Å) were observed, revealing the isolated dispersion of Au atoms. The peak from the Au-Cl shell was not observed in XAFS spectra, indicating that residual Cl from the synthesis was absent on Au_1_/BP nanosheets. This result was also supported by the high-resolution X-ray photoelectron spectroscopy (XPS) spectrum which showed no signals for Cl (Supplementary Fig. 7). Further density functional theory (DFT) calculations were performed to establish the atomic model of Au_1_/BP nanosheets. To simulate Au_1_/BP nanosheets, we situated the Au single atom at the bridge P sites (inset of Fig. 1d), considering thermodynamic stability and two Au–P bonds as revealed by EXAFS.

To explore the band structure of BP and Au_1_/BP nanosheets, we conducted measurements of ultraviolet-visible-near infrared (UV-vis-NIR) absorption and valence XPS. From UV-vis-NIR spectra and the corresponding Tauc plots, the bandgaps of BP and Au_1_/BP nanosheets were 1.45 eV (Supplementary Fig. 8a, b). Mott–Schottky plots showed that the flat band potentials of BP and Au_1_/BP nanosheets were both –0.35 V (vs RHE) (Supplementary Fig. 8c). According to valence XPS spectra, the valence band energy levels of BP and Au_1_/BP nanosheets were estimated as 1.35 and 1.31 eV below the Fermi level, respectively (Supplementary Fig. 8d). Based on UV-vis-NIR absorption and valence XPS spectra, the complete band structure of Au_1_/BP nanosheets was obtained, similar to that of BP nanosheets (Supplementary Fig. 8e). Therefore, the deposition of Au atoms on BP nanosheets led to negligible variation in the band structures of BP nanosheets. For comparison, we prepared Pt, Pd, and Rh single atoms/NPs on BP nanosheets, which were denoted as Pt_1_/BP, Pd_1_/BP, Rh_1_/BP, Pt NPs/BP, Pd NPs/BP, Rh NPs/BP nanosheets, respectively (Supplementary Figs. 9 and 10). The loading amounts of metal single atoms and NPs were controlled as 0.2 wt% and 1.0 wt%, respectively. The dispersion of metal species in these samples was further verified by diffuse reflectance infrared Fourier transform spectroscopy (DRIFTS) experiments using CO as a probe molecule. Especially, DRIFTS spectra of M_1_/BP (M = Pt, Pd, and Rh) nanosheets only showed the peaks for the linear adsorption of CO, whereas, besides the peaks for the linear adsorption, the peaks for the bridge adsorption were also observed for M NPs/BP nanosheets (Supplementary Fig. 11 and Table 2).

### Catalytic properties of Au_1_/BP nanosheets towards partial oxidation of methane

**Fig. 2:**
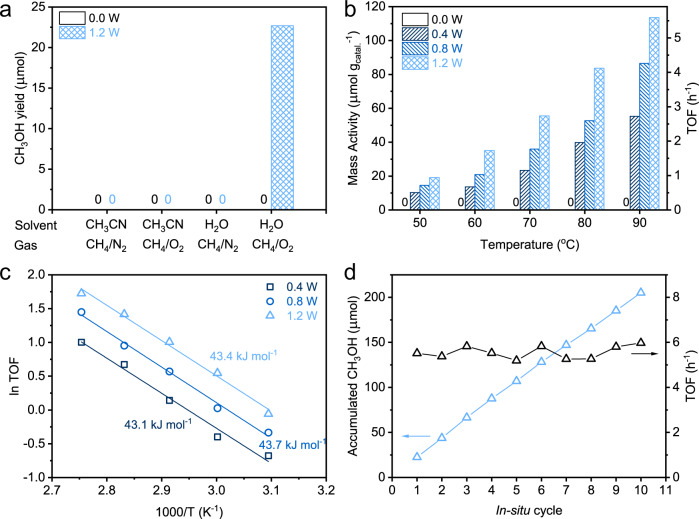
Catalytic performance of Au_1_/BP nanosheets in partial oxidation of methane. **a** Comparison of methanol yields under different solvents and reactants at 90°C for 2 h. **b** Mass activities and TOF numbers in 20 mL of water under 33 bar (CH_4_:O_2_ = 10:1) at different temperatures under different light powers with an irradiation area of 3.14 cm^2^. **c** The Arrhenius plots of Au_1_/BP nanosheets under different light intensities. **d** Products and TOF numbers obtained by conducting in situ cycles. For each cycle, the catalytic reaction was operated under 33 bar (CH_4_:O_2_ = 10:1) at 90°C for 2 h (1.2 W).

The catalytic tests were conducted in a slurry reactor with a sapphire window, which allowed the incidence of light from Xe lamp into the reactor to participate in the reaction (Supplementary Fig. 12). For a standard condition, the reaction was operated in 20 mL of water pressured with 33 bar of mixed gas (CH_4_:O_2_ = 10:1) at 90 °C under light irradiation with a light power of 1.2 W and irradiation area of 3.14 cm^2^. For BP nanosheets, the product after 2 h was below detection limit. As for Au_1_/BP nanosheets, 22.7 μmol of methanol was generated without any by-products after 2 h (Fig. 2a). Under the standard condition, the mass activity of Au_1_/BP nanosheets was 113.5 μmol g_catal_^−1^, whereas the turnover frequency (TOF) number was 5.6 h^−1^. Notably, the TOF number of Au_1_/BP nanosheets was higher than those of Au NPs/BP, M_1_/BP, and M NPs/BP (M = Pt, Rh, and Pd) nanosheets (Supplementary Fig. 13). The TOF number of Au_1_/BP nanosheets was comparable to that of the state-of-the-art catalysts under similar reaction conditions (Supplementary Table 3). When we removed light irradiation from the standard condition, the product was below detection limit (Fig. 2a). As such, light irradiation helped drive the formation of methanol.

To further investigate the influence of light irradiation, we varied the light powers from 0.4 to 1.2 W. As shown in Fig. 2b and Supplementary Figure 14, the activity of Au_1_/BP nanosheets increased with the rise of light intensity at the same temperature. Notably, the activation energy (*E*_a_) of Au_1_/BP nanosheets was almost independent of light intensity (Fig. 2c). Especially, *E*_a_ of Au_1_/BP nanosheets at the power of 1.2 W was 43.4 kJ mol^−1^, approaching to that (43.7 kJ mol^−1^) at 0.8 W and that (43.1 kJ mol^−1^) at 0.4 W (Fig. 2c). In addition, the reaction was determined as heterogeneous instead of homogeneous, because the use of the supernate after the reaction, HAuCl_4_-H_3_PO_4_, and Au(OH)_3_-H_3_PO_4_ as the catalysts gave rise to trace amounts of products (Supplementary Fig. 15). Moreover, we evaluated the wavelength-dependent apparent quantum yields (AQYs) of methanol. A high AQY of 17.4% was obtained under the irradiation of monochromatic light at 350 nm (Supplementary Fig. 16).

We further explored the influence of the solvent and reactants during partial oxidation of methane. Substitution of water with acetonitrile as the solvent from the standard condition gave rise to no products (Fig. 2a). In this case, water enables mild oxidation of methane under light irradiation. When O_2_ was replaced by N_2_ from the standard condition, leaving water as the oxidant, no detectable product was observed (Fig. 2a). As such, O_2_ was an indispensable oxidant, whereas only water was unable to oxidize methane under such mild reaction. In order to investigate the dependence of catalytic performance on reactants, we varied the partial pressures of CH_4_ and O_2_ at 90 °C under light irradiation. Increasing CH_4_ partial pressure led to the rise of conversion while keeping the selectivity for methanol above 99% (Supplementary Fig. 17a). Increasing O_2_ partial pressure also increased the conversion and lowered the selectivity for methanol with the production of CO_2_ (Supplementary Fig. 17b). To determine whether the reaction was limited by mass transfer, we varied the stirring rates and the volumes of water. Despite of the varied stirring rates and the volumes of water, the mass activity still kept almost unchanged, indicating that the reaction kinetics was not determined by diffusion (Supplementary Fig. 18). When we used methanol as the reactants over Au_1_/BP nanosheets under light irradiation and 33 bar (N_2_:O_2_ = 10:1) at 90 °C, the conversion of methanol was below detection limit. In this case, Au_1_/BP nanosheets were unable to catalyze the oxidation of methanol under such reaction conditions, indicating the high intrinsic selectivity for methanol.

To investigate stability of the catalysts, we tested Au_1_/BP nanosheets for successive in situ cycles of reaction, where catalysts were not removed from the reaction during the whole test. For each cycle, the catalytic reaction proceeded under standard conditions for 2 h. After 10 in situ cycles (20 h in total), about 205.2 μmol of methanol was generated in total with the fluctuation of TOF below 7% (Fig. 2d). Moreover, the atomic dispersion of Au was still maintained after 10 in situ cycles, where only Au–P bonds were observed in the absence of Au–Au bonds or Au–O bonds (Supplementary Fig. 19 and Table 1).

### Mechanistic insights into the activation of O_2_

**Fig. 3:**
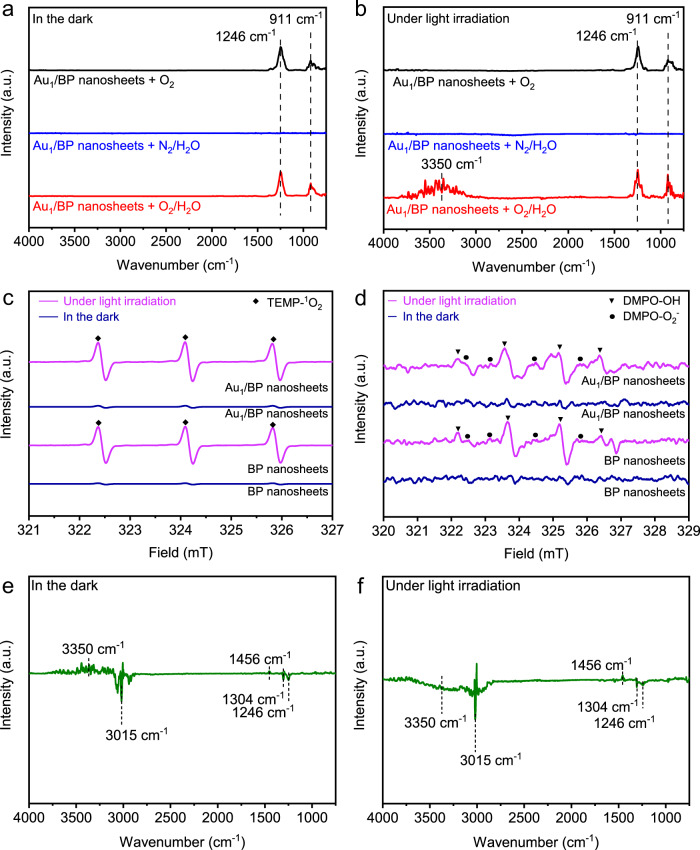
Mechanistic studies. In situ DRIFTS spectra of Au_1_/BP nanosheets purged by 1 bar of different gases at 90°C (**a**) in the dark and (**b**) under light irradiation. Background spectra were acquired after flowing under 1 bar of N_2_ with the rate of 20 sccm at 150°C for 0.5 h, followed by cooling to 90°C. **c** In situ ESR spectra of BP and Au_1_/BP nanosheets under different conditions in the presence of TEMP. **d** In situ ESR spectra of BP and Au_1_/BP nanosheets under different conditions in the presence of DMPO. In situ DRIFTS spectra of Au_1_/BP nanosheets purged by 1 bar of CH_4_ at 90°C (**e**) in the dark and (**f**) under light irradiation. Catalysts were purged by 1 bar of O_2_/H_2_O at 90°C for 0.5 h, followed by 1 bar of N_2_ at 150°C for 0.5 h. Afterwards, the catalysts were purged by 1 bar of CH_4_ at 25°C for 0.5 h to obtain background spectra.

In order to investigate how water promotes methane oxidation under light irradiation, we conducted in situ DRIFTS measurements. Background spectra were acquired after flowing with 1 bar of N_2_ at 150 °C for 0.5 h, followed by cooling to 90 °C. In situ DRIFTS spectrum of Au_1_/BP nanosheets after the treatment with O_2_ in the dark at 90 °C showed two peaks at 1246 and 911 cm^−1^, which were assigned to the stretching vibrations of P=O and P–O–P bonds, respectively (Fig. 3a). The assignment of the peaks was supported by DFT calculations, reported literature, and isotope-labeling DRIFTS measurements (Supplementary Table 4 and Fig. 20). The peaks for P=O and P–O–P bonds were also observed when Au_1_/BP nanosheets were purged by O_2_ under light irradiation (1.2 W) at 90 °C (Fig. 3b). To simulate the reaction condition in the presence of water, N_2_ was allowed to bubble in deionized water, followed by flowing into the cell, in order to bring the saturated water vapor into the cell, denoted as the treatment of N_2_/H_2_O. Under the atmosphere of N_2_/H_2_O, no peaks were observed, whenever in the dark or under light irradiation (Fig. 3a, b). As such, O_2_ enabled the oxidization of BP nanosheets, whereas only H_2_O interacted weakly with the catalyst. Compared with the circumstances of O_2_, the mixture of O_2_/H_2_O made negligible differences in the dark, but gave rise to a new peak at 3350 cm^−1^ for the stretching vibration of hydroxyl groups under light irradiation (Fig. 3a, b). In this case, water reacted with O_2_ over Au_1_/BP nanosheets under light irradiation, resulting in the formation of hydroxyl groups.

We further explored whether hydroxyl groups were bonded with P atoms or Au atoms. We conducted quasi situ XPS measurements after the treatment of Au_1_/BP nanosheets with O_2_/H_2_O mixed gas in the dark or under light irradiation at 90 °C. O 1 *s* spectra confirmed that P–O–P and P=O bonds were formed in the dark, whereas P–OH bonds were generated under light irradiation (Supplementary Fig. 21a). Whenever Au_1_/BP nanosheets were treated with O_2_/H_2_O in the dark or under light irradiation, Au 4 *f* spectra showed negligible difference compared with that without any treatment (Supplementary Fig. 21b). In this case, Au atoms were not further oxidized by O_2_/H_2_O in terms of the coordination with oxygen-containing groups such as O atoms or hydroxyl groups. To further verify this point, we conducted ^1^H solid nuclear magnetic resonance (NMR) measurements with the reference of Au(OH)_3_. After the treatment of Au_1_/BP nanosheets with O_2_/H_2_O under light irradiation, magic angle spinning (MAS) NMR spectrum showed no detectable signals (Supplementary Fig. 22), excluding the possibility of the adsorption of hydroxyl groups on Au atoms. Therefore, hydroxyl groups were adsorbed on P atoms, generating P–OH ensembles.

Considering the short lifetime of photo-induced radicals that cannot be detected by in situ DRIFTS or quasi situ XPS, we conducted measurements of in situ electron spin resonance (ESR) spectroscopy using different trapping agents. 2,2,6,6-tetramethylpiperidine (TEMP) was selected to detect singlet O_2_ (^1^O_2_), while 5,5-dimethyl-1-pyrroline-N-oxide (DMPO) was selected for the detection of superoxide ions (O_2_^−^) and hydroxyl radicals (•OH). As illustrated in Figure 3c, d, signals for ^1^O_2_, O_2_^−^, and •OH were all observed for both BP and Au_1_/BP nanosheets under light irradiation. Collectively, the water-promoted activation of O_2_ under light irradiation involves the formation of ^1^O_2_, O_2_^−^, •OH, and P–OH species.

We carried out DFT calculations to determine the adsorption configurations of dissociated O atoms or hydroxyl groups on Au_1_/BP nanosheets. The optimized configurations were obtained according to thermodynamic consideration. Supplementary Figure 23 shows the optimized adsorption configuration of two O atoms on Au_1_/BP nanosheets. Especially, one O atom (O1) is located at the top P atom to form a P=O ensemble. The other O atom (O_2_) at the groove site is linked to two P atoms to form a P–O–P ensemble. These two ensembles were both observed in in situ DRIFTS spectrum (Fig. 3a). As such, the activation of O_2_ in the dark follows Eq. 4.

4 $${\mathrm{O}}_2 + 3{\mathrm{P}} \to {\mathrm{P}} = {\mathrm{O}} + {\mathrm{P}} - {\mathrm{O}} - {\mathrm{P}}$$

**Fig. 4:**
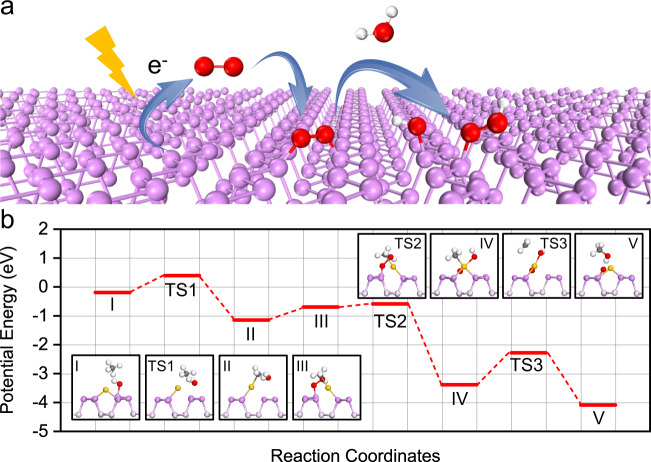
DFT studies. **a** Schematic illustration of oxygen activation on BP nanosheets. **b** Reaction path for partial oxidation of methane over Au_1_/BP nanosheets under light irradiation. The inset images show the side views of the configurations. Yellow, violet, pink, red, gray, and white spheres represent Au, surface P, subsurface P, O, C, and H atoms, respectively.

Figure 4a shows the activation of O_2_ under light irradiation (Supplementary Fig. 24). Especially, the electrons from the valence band of BP are excited by light irradiation to react with O_2_ at the triplet ground states (^3^O_2_), resulting in the formation of O_2_^−^ (Eq. 5). O_2_^−^ recombines the photo-generated holes to form the excited O_2_ at the singlet states, ^1^O_2_ (Eq. 6). ^1^O_2_ reacts with H_2_O to yield OH^*^ and HOO^*^ which can be split into •OH radicals (Eqs. 7 and 8).

5 $${\,}^3{\mathrm{O}}_2 + e^ {-} \to {\mathrm{O}}_2^ {-}$$

6 $${\mathrm{O}}_2^ - + h^ + \to {\,}^1{\mathrm{O}}_2$$

7 $$^1{\mathrm{O}}_2 + {\mathrm{H}}_2{\mathrm{O}} \to {\mathrm{HOO}}^ \ast + {\mathrm{OH}}^ \ast$$

8 $${\mathrm{HOO}}^ \ast \to \bullet \,{\mathrm{OH}} + {\mathrm{O}}^ \ast$$

### Mechanistic insights into the oxidation of CH_4_

To reveal the role played by P–OH ensembles, we carried out in situ DRIFTS measurements of Au_1_/BP nanosheets under CH_4_ after forming hydroxyl groups or not. Especially, the catalyst was purged by 1 bar of O_2_/H_2_O at 90 °C for 0.5 h in the dark to generate P=O and P–O–P ensembles, or under light irradiation to yield P=O, P–O–P, and P–OH ensembles. Afterwards, the catalyst was purged by 1 bar of CH_4_ at 25 °C for 0.5 h to obtain background spectra. In situ DRIFTS spectra were recorded with CH_4_ flow at 90 °C in the dark or under light irradiation. Notably, in in situ DRIFTS spectra, upward peaks correspond to newly appeared species, whereas downward peaks correspond to the loss of existed species.

When both the pre-treatment and in situ DRIFTS measurements were conducted in the dark, both upward and downward peaks were observed (Fig. 3e). Upward peaks included that at 3350 cm^−1^ for P–OH ensembles and that at 1456 cm^−1^ for CH_3_^*^. Downward peaks included those at 3015 and 1304 cm^−1^ for CH_4_^*^, and that at 1246 cm^−1^ for P=O ensembles. As such, P–OH and CH_3_^*^ was generated, whereas P=O ensembles and CH_4_^*^ were consumed. We propose that the reaction occurred as Eq. 9.

9 $${\mathrm{CH}}_4^ \ast + {\mathrm{P}} = {\mathrm{O}} \to {\mathrm{CH}}_3^ \ast + {\mathrm{P}} - {\mathrm{OH}}$$

Considering the absence of products in the dark, CH_3_^*^ was unable to be further oxidized into methanol under mild condition in the dark. In other words, partial dehydrogenation of methane yielded inert P–OH ensembles.

When both the pre-treatment and in situ DRIFTS measurements were conducted under light irradiation, the downward peaks comprised those for P–OH ensembles, P=O ensembles, and CH_4_^*^, whereas the upward peak only contained that for CH_3_^*^ (Fig. 3f). Considering methanol production under light irradiation, such reactive P–OH ensembles allowed mild oxidation of methane into CH_3_^*^ and methanol in sequence. In addition, the in situ DRIFTS spectrum recorded in the dark after the pre-treatment under light irradiation was similar to Fig. 3f (Supplementary Fig. 25). To further identify how P–OH ensembles participated in the reaction, we combined temperature-programmed surface reaction with mass spectroscopy (TPSR-MS) using an isotope label. Especially, we used ^18^O-labeled water (H_2_^18^O) and routine O_2_ (^32^O_2_) to pre-treat the catalyst in the dark or under light irradiation. TPSR-MS measurements were operated in the dark under CH_4_ when the temperature rose from 50 to 300 °C. For pre-treatment in the dark, all the products were below the detection limit (Supplementary Fig. 26a, b). With regard to pre-treatment under light irradiation, four types of products were detected, including CH_3_^18^OH, CH_3_^16^OH, H_2_^18^O, and H_2_^16^O (Supplementary Fig. 26c). Moreover, no peaks for CO_2_ were observed, indicating the high selectivity for methanol (Supplementary Fig. 26d). Notably, the temperature (above 200 °C) for methanol production was much higher than that (90 °C) during catalytic tests under light irradiation, because TPSR was conducted in the dark. In other words, methane oxidation under light irradiation follows a different path from that in the dark, despite O_2_ activation under light irradiation.

To investigate the mechanisms of methane oxidation under different conditions, we conducted DFT calculations. When both O_2_ activation and CH_4_ oxidation proceed in the dark, CH_4_ dissociates into CH_3_^*^ bonded to an Au atom and H^*^ stabilized by a P=O ensemble to form a P–OH ensemble (Supplementary Fig. 27). The reaction of P–OH with CH_3_^*^ exhibits an energy difference between configurations II and TS3 of 2.18 eV, which is much higher than that (0.42 eV, configurations II → TS1) of the recombination of CH_3_^*^ and H^*^ into CH_4_ (Supplementary Fig. 27). The apparent energy barrier is defined as the energy difference between the initial state and the transition state with the highest energy. In this case, the apparent energy barrier (configurations I → TS3) is 2.62 eV for the conversion of CH_4_ into CH_3_OH. Therefore, in the dark, P–OH ensembles cannot afford further oxidation of CH_3_^*^ into methanol over Au_1_/BP nanosheets, though both O_2_ and CH_4_ can be activated.

After the activation of O_2_ under light irradiation, we consider the oxidation of CH_4_ in the dark. The lack of continuous light leads to the adsorption of •OH into hydroxyl groups. Once two reactive hydroxyl groups are formed, one hydroxyl group reacts with CH_4_ to generate CH_3_^*^ and water. This step is the most thermodynamically favorable among different routes of CH_4_ activation, exhibiting an energy barrier of 0.58 eV (Supplementary Table 5 and Fig. 28, configurations I → TS1). Afterwards, the other hydroxyl group migrates from P atom to Au atom for further oxidation of CH_3_^*^ (Supplementary Fig. 28, configurations III → TS2 → IV). The last step is the integration of hydroxyl groups and CH_3_^*^ to form methanol (Supplementary Fig. 28, configurations IV → TS3 → V). The barrier of this step is 1.15 eV, thus determined as the rate-limiting step. Notably, the energy difference between configurations III and TS3 is as large as 1.67 eV (Supplementary Fig. 28). The apparent energy barrier (configurations I → TS3) is 1.03 eV for the conversion of CH_4_ into CH_3_OH (Supplementary Fig. 28).

We further focused on the reaction channel when both O_2_ activation and CH_4_ oxidation proceed under light irradiation. Upon the activation of O_2_, the generated hydroxyl group reacts with CH_4_ to form water and CH_3_^*^ stabilized by an Au atom (Fig. 4b). The breakage of O–O bond in HOO^*^ generates •OH which further co-adsorbs and reacts with CH_3_^*^ on the Au atom, exhibiting an energy barrier as low as 0.25 eV (Fig. 4b, configurations III → TS2). Methanol is produced by the combination of OH^*^ and CH_3_^*^ on the Au atom with the energy barrier of 1.10 eV, which is determined as the rate-limiting step (Fig. 4b, configurations IV → TS3 → V). Notably, the remaining oxygen atoms on Au_1_/BP nanosheets are also able to activate CH_4_ at 90 °C with the energy barrier of 1.19 eV, so as to complete the reaction cycle (Supplementary Fig. 29). Moreover, the apparent energy barrier for CH_4_ conversion into CH_3_OH under light irradiation (Fig. 4b, configurations I → TS1) is only 0.58 eV, much lower than that (1.03 eV) in the dark. These results explain that 90 °C enables the oxidation of methane into methanol during catalytic tests under light irradiation, whereas methanol was produced above 200 °C during TPSR-MS experiments after the pre-treatment under light irradiation.

To explain the high selectivity for methanol, we investigated the processes of CH_4_ dehydrogenation and methanol oxidation. Except for CH_4_ dehydrogenation into CH_3_^*^, the other dehydrogenation steps (CH_x_^*^ → CH_x−1_^*^ + H^*^, *x* = 1, 2, 3) are all highly endothermic (Supplementary Fig. 30). In this case, the Au atom stabilizes CH_3_* to prevent deeper dehydrogenation. Moreover, we weighed the energy barriers for methanol oxidation, methanol desorption, and methane activation. Especially, the energy barrier for methanol oxidation by P–OH species is 1.31 eV, which is higher than that (apparent energy barrier, 0.58 eV) for methane activation and that (0.90 eV) for methanol desorption (Supplementary Fig. 31). Similar case also applies to P=O species where the energy barriers for methanol oxidation and desorption are 1.82 and 0.83 eV, respectively (Supplementary Fig. 31). As such, the produced methanol tends to desorb from the catalyst surface and dissolve in water instead of further oxidation under mild conditions such as 90 °C and 3 bar of O_2_ partial pressure. Moreover, considering the low concentration of the produced methanol and the stabilization of methanol by the polar solvent of water, the dissolved methanol is unlikely to re-adsorb on the catalyst surface for further oxidation.

In conclusion, we demonstrated that water enabled mild oxidation of methane into methanol with >99% selectivity over Au_1_/BP nanosheets under light irradiation. We found that water and O_2_ reacted under light irradiation to generate reactive hydroxyl groups and •OH radicals. Hydroxyl groups reacted with methane at Au single atoms to form water and CH_3_^*^ species. CH_3_^*^ species were further oxidized by •OH radicals to generate methanol. As water is completely recycled during the whole process, it can also be regarded as a catalyst. Our findings shed light on insights into the role played by water during methane oxidation. Moreover, this work offers an effective strategy for upgrading methane at ambient condition suitable for local, on-site applications.

## Methods

### Catalyst preparation

In all, 500 mg of red phosphorus, 20 mg of Sn, and 10 mg of SnI_4_ were sealed in an evacuated Pyrex tube. The tube was heated to 650 °C with a rate of 1.4 °C min^−1^ and kept at 650 °C for 5 h, followed by cooling down to 500 °C with a rate of 0.3 °C min^−1^. After the sample, was naturally cooled down to room temperature, the product was washed with hot toluene and acetone for three times to remove the residual mineralizer. Finally, the product was dried under vacuum for further analysis. BP nanosheets were prepared by liquid exfoliation of bulk BP. In detail, 200 mg of bulk BP was dispersed in 500 mL of NMP. The mixture solution was then sonicated in ice water for 8 h. Ice water was used to keep the system at relatively low temperature. Afterwards, the resultant brown suspension was centrifuged at 150 × *g* for 10 min to remove the residual unexfoliated particles. The supernatant was collected by centrifugation at 11,242 × *g* for 10 min and washed by a mixture of hexane, chloroform, and ethanol. After been washed for three times, BP nanosheets were dried under vacuum for further use. In a typical synthesis of Au_1_/BP nanosheets, 400 mg of BP nanosheets was finely dispersed in 500 mL of ethanol under N_2_ protection. Then, 20 mL of HAuCl_4_ aqueous solution (0.203 mM) was injected into the solution containing BP nanosheets through a two-channel syringe pump at room temperature for 4 h under N_2_ protection. The product was collected by centrifugation, washed three times with ethanol, and then dried under vacuum. Further ICP-AES result determined that the mass loading of Au was 0.2 wt%. The syntheses of Pt_1_/BP, Rh_1_/BP, and Pd_1_/BP nanosheets with metal loading of 0.2 wt% followed the same method as that of Au_1_/BP nanosheets, despite that H_2_PtCl_4_, RhCl_3_, and Na_2_PdCl_4_ were used as precursors, respectively. The syntheses of Au NPs/BP, Pt NPs/BP, Rh NPs/BP, and Pd NPs/BP nanosheets with metal loading of 1.0 wt% followed the method of NaBH_4_ reduction. In all, 400 mg of BP nanosheets were finely dispersed in 500 mL of ethanol under N_2_ protection. Then, 20 mL of HAuCl_4_ (1.02 mM), H_2_PtCl_4_ (1.03 mM), RhCl_3_ (1.94 mM), or Na_2_PdCl_4_ (1.88 mM) aqueous solution was added into BP nanosheets suspension and stirred for 20 min under N_2_ protection. In all, 8 mL of NaBH_4_ aqueous solution (20 mg/mL) was added into the mixture. After further stirring for 40 min, the samples were collected by centrifugation, washed with ethanol for three times, and then dried under vacuum.

### Catalytic tests

The partial oxidation of methane into methanol was carried out in a 180-mL stainless-steel slurry reactor (Shanghai Yanzheng Experiment Instrument Co., LTD, China). The sapphire window with an irradiation area of 3.14 cm^−2^ at the top of slurry reactor allows the incidence of light from Xe lamp (PLS-SXE300, PerfectLight, China) into the reactor to participate in the photocatalysis. Full-spectrum light was adopted for catalytic tests. The intensity of irradiation light was determined by a spectroradiometer (PL-MW2000, PerfectLight, China). After the addition of 20 mL of H_2_O and 200 mg of catalysts into a Teflon inlet, the slurry reactor was pressurized with CH_4_ (30 bar) and O_2_ (3 bar) at room temperature. Then, the slurry reactor was heated to 90 °C and kept for 2 h with stirring at 500 rpm. After completion of the reaction, the slurry reactor was quickly cooled by ice water. The gas-phase was determined by gas chromatographs (Shimadzu GC-2014) equipped with a thermal conductivity detector (TCD) and a flame ionization detector (FID). The liquid phase of the reaction mixture was collected by centrifugation at 14,962 × *g* for 5 min. 20 μL of DMF was introduced into 0.4 mL of the reaction mixture as an internal standard. In all, 100 μL of the mixture was dissolved in 0.4 mL of DMSO-d_6_ to determine the product yields by ^1^H NHR spectroscopy.

### In situ cycles

For each cycle, the catalytic reaction was operated under CH_4_ (30 bar) and O_2_ (3 bar) at 90 °C for 2 h. After cooling down, the product in gas phase was detected by both GC-TCD and GC-FID. 0.4 mL of liquid products were taken for further analysis without open the reactor. Afterwards, the reactor was re-pressured with CH_4_ (30 bar) and O_2_ (3 bar), followed by being reheated to 90 °C and kept at this temperature for another 2 h. The whole durability test continued for 10 in situ cycles.

### XAFS measurements

XAFS spectra at Au L_3_-edge (*E*_0_ = 11,919 eV) were performed at BL14W1 beamline of Shanghai synchrotron radiation facility operated at 3.5 GeV under “top-up” mode with a constant current of 220 mA. The XAFS data were recorded under fluorescence mode with a 3_2_^−^element Ge solid state detector. The energy was calibrated according to the absorption edge of pure Au foil. Athena and Artemis codes were used to extract the data and fit the profiles. For the XANES part, the experimental absorption coefficients as a function of energies *μ*(*E*) were processed by background subtraction and normalization procedures. This process was reported as “normalized absorption”. For the EXAFS part, the Fourier transformed (FT) data in *R* space were analyzed by applying the first shell approximation or metallic Au model for the Au–P or Au–Au shell, respectively. The passive electron factors, *S*_0_^2^, were determined by fitting the experimental Au foil data and fixing the Au–Au *CN* to be 12, and then fixed for further analysis of the measured samples. The parameters describing the local structure environment including *CN*, bond distance (*R*) and Debye Waller (*D.W*.) factor around the absorbed atoms were allowed to vary during the fit process.

### In situ DRIFTS measurements

In situ DRIFTS experiments were conducted in a cell with a Fourier transform infrared spectrometer and a liquid nitrogen cooled detector. In all, 1 mg of Au_1_/BP was diluted in 200 mg of KBr.

As for treatments without CH_4_ in the dark (Fig. 3a), the background spectrum of the sample was acquired after flowing under 1 bar of N_2_ with the rate of 20 sccm at 150 °C for 0.5 h, followed by cooling to 90 °C. For the treatment with O_2_, the sample was purged by 1 bar of O_2_ with the rate of 10 sccm at 90 °C for 0.5 h, followed by 1 bar of N_2_ with the rate of 20 sccm at 90 °C for 0.5 h. The procedure for the treatment with N_2_ was similar to that for the O_2_ treatment, except for the use of 1 bar of N_2_ to replace O_2_. For the treatment with O_2_/H_2_O, 1 bar of O_2_ was allowed to bubble in deionized water, followed by flowing into the cell, in order to bring the saturated water vapor into the cell. The sample was purged by the mixed gas containing O_2_ and H_2_O with the rate of 10 sccm at 90 °C for 0.5 h, followed by 1 bar of N_2_ with the rate of 20 sccm at 90 °C for 0.5 h. The procedure for the treatment with N_2_/H_2_O was similar to that for the treatment with O_2_/H_2_O, except for the use of 1 bar of N_2_ to replace O_2_.

The procedures for treatments without CH_4_ under light irradiation (Fig. 3b) were similar to those in the dark, except for using Xe lamp (1.2 W) to illuminate the cell.

As for treatments with CH_4_ in the dark (Fig. 3e), the sample was pre-treated under 1 bar of O_2_/H_2_O with the rate of 10 sccm at 90 °C for 0.5 h to generate P=O and P–O–P ensembles. Afterwards, the sample was purged by 1 bar of CH_4_ with the rate of 10 sccm at 25 °C for 0.5 h to obtain background spectra. In situ DRIFTS spectrum was recorded under 1 bar of CH_4_ with the rate of 10 sccm at 90 °C in the dark.

The procedure for the treatment with CH_4_ under light irradiation (Fig. 3f) was similar to that in the dark, except for using Xe lamp (1.2 W) to illuminate the cell. Notably, the pre-treatment with O_2_/H_2_O under light irradiation aimed at yielding P=O, P–O–P, and P–OH ensembles.

### DRIFTS measurements using CO as a probe molecule

The background spectrum of the sample was acquired after flowing under 1 bar of N_2_ with the rate of 20 sccm at 150 °C for 0.5 h, followed by cooling to 25 °C. Then, 1 bar of 10%CO/Ar with the rate of 10 sccm was allowed to flow into the cell for 20 min. 1 bar of N_2_ with the rate of 20 sccm was used to purge out the gaseous CO from the sample cell so that the chemically adsorbed CO species on the samples could be detected.

### In situ ESR measurements

Au_1_/BP nanosheets were dispersed in water pre-saturated with CH_4_ and O_2_ to detect possible radicals (4 g L^−1^). As for the ^1^O_2_ trapping-ESR tests, 50 μL of aqueous suspension of samples was mixed with 500 μL of 2,2,6,6-tetramethylpiperidine (50 mM) solution. After being illuminated for 1 min, the mixture was characterized using an ESR spectrometer (JEOL JES-FA200) at room temperature. As for the O_2_^−^ and •OH trapping-ESR tests, similar procedures were adopted except the use of 5,5-dimethyl-1-pyrroline-N-oxide (DMPO) as the spin-trapping agent.

### TOF calculation

The TOF numbers were calculated based following equation (equation 10):

10 $$\begin{array}{l}{\mathrm{TOF}}\\ = \left( {n_{{\mathrm{CH}}3{\mathrm{OH}}} + n_{{\mathrm{CO}}2}} \right)/\left( {t \times n_{{\mathrm{surface}}}} \right)\\ = \left( {n_{{\mathrm{CH}}3{\mathrm{OH}}} + n_{{\mathrm{CO}}2}} \right)/\left( {t \times \delta \times n_{{\mathrm{metal}}}} \right)\\ = \left( {n_{{\mathrm{CH}}3{\mathrm{OH}}} + n_{{\mathrm{CO}}2}} \right){\mathrm{ }}/{\mathrm{ }}\left( {t \times \delta \times m_{{\mathrm{cat}}} \times w/\mu _{{\mathrm{metal}}}} \right)\end{array}$$

In these equations, *n*_CH3OH_ represents the mole of produced CH_3_OH molecules. *t* is the reaction time. *n*_surface_ is the mole of surface metal atoms. *δ* is the molar ratio of surface atom. *n*_metal_ is the mole of total metal atoms. *m*_cat_ is the mass of the catalysts. *w* is the mass loading of the catalysts. *μ*_*metal*_ is the atomic mass. For single atoms, *δ* = 100%. For NPs, *δ* was calculated using the following equations (equations 11–13) given by Vannice^29^:

11 $$\begin{array}{l}\delta = 6 \times 10^7 \times \left( {V_{{\mathrm{metal}}}/A_{{\mathrm{metal}}}} \right) \times \left( {1/d_{{\mathrm{metal}}}} \right) \times 100\% \\ \end{array}$$

12 $$V_{{\mathrm{metal}}} = \mu _{{\mathrm{metal}}}/\left( {\rho _{{\mathrm{metal}}} \times N_A} \right)$$

13 $$A_{{\mathrm{metal}}} = 1/n_s$$

where *V*_metal_ is the bulk atomic volume of the metals (cm^3^), *A*_metal_ is the area of an atom (cm^2^), and *d* is the metal particle size in nm. *μ*_metal_ is the atomic mass, *ρ* is the bulk density, and *N*_A_ is Avogadro’s number. *n*_s_ is the average site density. The *n*_s_ of Au, Pt, Rh, Pd were 1.2 × 10^15^, 1.3 × 10^15^, 1.3 × 10^15^, and 1.3 × 10^15^ cm^−2^, respectively^30^. Thus *A*_metal_ were calculated as 8.7 × 10^−16^, 8.0 × 10^−16^, 7.5 × 10^−16^, 7.9 × 10^−16^ cm^2^. The values of *V*_metal_ were calculated as 1.7 × 10^−23^, 1.5 × 10^−23^, 1.4 × 10^−23^, 1.5 × 10^−23^ cm^3^ for Au, Pt, Rh, and Pd, respectively. The average sizes (*d*_metal_) of NPs were measured to be 6 nm, 5 nm, 4 nm, and 6 nm for Au, Pt, Rh, and Pd, respectively, based on TEM images. Therefore, the values of *δ* were calculated to be 19.5%, 22.6%, 27.5%, and 18.6% for Au, Pt, Rh, and Pd, respectively. The TOF numbers of Au_1_/BP, Pt_1_/BP, Rh_1_/BP, Pd_1_/BP, Au NPs/BP, Pt NPs/BP, Rh NPs/BP, and Pd NPs/BP nanosheets were calculated as 5.6, 4.5, 1.1, 3.2, 3.3, 0.8, 0.2, and 2.1 h^−1^, respectively.

### Conversion calculation

The conversion was calculated based following equation (equation 14):

14 $${\mathrm{Conversion}} = \left( {n_{{\mathrm{CH}}3{\mathrm{OH}}} + n_{{\mathrm{CO}}2}} \right)/n_{{\mathrm{CH}}4} \times 100\%$$

The amount of CH_4_ charged into the reactor is calculated from 30 bar of CH_4_ at room temperature. The volume of gas is 160 mL, which corresponds to 193.6 mmol CH_4_. The conversion after 2 h was calculated as 0.01%. We chose a low conversion in order to measure the kinetic parameters such as the reaction rate, TOF, and *E*_a_, because the temperature, pressure, and concentrations can be regarded as constant throughout the reactor^31^.

### AQY measurements

The apparent quantum yields (AQY) were measured under the irradiation of monochromatic lights at 350, 380, 450, 500, 550, 600, 650, and 765 nm using band-pass filters. The irradiation area was 3.14 cm^2^. The intensities of irradiation light was determined to be 2.1, 4.2, 21.9, 15.6, 19.3, 23.9, 26.0, and 23.9 mW·cm^−2^ for 350, 380, 450, 500, 550, 600, 650, and 765 nm, respectively. The formation of one methanol molecule only consumes one photo-generated electron during the generation of reactive hydroxyl groups and •OH radicals. The AQY was calculated according to the following equation (equation 15):

15 $${\mathrm{AQY}} =	\, 1 \times N_{{\mathrm{CH}}3{\mathrm{OH}}}/N_{{\mathrm{photons}}} \times 100\% \\ =	\, 1 \times N_{{\mathrm{CH}}3{\mathrm{OH}}} \times N_A/[W \times A \times t/(h \times \upsilon )] \times 100\%$$

*N*_CH3OH_ and *N*_photons_ represent the number of formed CH_3_OH and the number of incident photons. *υ*, *W*, *A*, and *t* are the incident light frequency, intensity, irradiation area and time, respectively. *N*_A_ and *h* are the Avogadro’s constant and Planck constant, respectively. The AQYs were calculated to be 17.4%, 8.7%, 1.3%, 0.9%, 1.0%, 0.6%, 0.4%, and 0.4% for 350, 380, 450, 500, 550, 600, 650, and 765 nm, respectively.

### TPSR-MS measurements

In all, 100 mg of Au_1_/BP nanosheets were pre-treated by being immersed in 5 mL ^18^O-labeled water (H_2_^18^O) under O_2_ (^32^O_2_) flow in the dark or under light irradiation (1.2 W) at 90 °C for 0.5 h. The treated samples were dried under vacuum, followed by being loaded into the quartz tube for further measurement. For TPSR-MS measurements, the pre-treated samples were heated in helium with a gas-flow rate of 20 mL min^−1^ at 200 °C for 1 h. After cooling down to 50 °C, the gas was switched to 10% CH_4_/Ar (v/v) with a gas-flow rate of 20 mL min^−1^. Mass spectral responses were collected from 50 to 300 °C at 5 °C min^−1^. We detected the mass numbers (m/z) of 33 for CH_3_^18^OH, 31 for CH_3_^16^OH, 19 for H_2_^18^O, 17 for H_2_^16^O, 48 for C^18^O_2_, 46 for C^18^O^16^O, and 44 for C^16^O_2_.

### DFT calculations

Spin-polarized DFT calculations were performed through Vienna ab initio simulation package^32,33^. The electron-ion interaction was described via the projector augmented wave method^34^. The exchange-correlation interaction was described via the optB86b-vdW functional^35^. The plane-wave basis set was used to solve the Kohn–Sham equations, whereas the kinetic energy cutoff was 400 eV. In our calculations, a 12-Å vacuum layer and a single layer of BP with (3 × 3) supercells were constructed to simulate Au_1_/BP nanosheets. All the atoms were relaxed during the structure optimization until the maximum force on any ion was less than 0.02 eV Å^−1^. The (4 × 3 × 1) *k*-point mesh was used to sample the Brillouin zone. The climbing-image nudged elastic band method was used to identify the transition states until the maximum force on any ion was less than 0.05 eV Å^−1^^36^.

## Supplementary information

Supplementary Information

## Data Availability

The data that support the findings of this study are available from the corresponding author upon reasonable request.

## Code availability

The code is available from the corresponding author upon reasonable request.
