# Supplementary material for: Water enables mild oxidation of methane to methanol on gold single-atom catalysts
Source: Nat Commun. 2021 Feb 22;12:1218. doi: 10.1038/s41467-021-21482-z (PMC7900127; doi:10.1038/s41467-021-21482-z)
Supplement: Supplementary file 1 — Supplementary Information [file 41467_2021_21482_MOESM1_ESM.pdf]

Supplementary Information for

Water enables mild oxidation of methane to methanol on gold single-atom catalysts

Laihao Luo[1], Jie Luo[1], Hongliang Li[1], Fangning Ren[1], Yifei Zhang[1], Andong Liu[1], Wei-Xue Li[1], Jie Zeng[1]

[1]Hefei National Laboratory for Physical Sciences at the Microscale, CAS Key Laboratory of Strongly-Coupled Quantum Matter Physics, Key Laboratory of Surface and Interface Chemistry and Energy Catalysis of Anhui Higher Education Institutes, Department of Chemical Physics, University of Science and Technology of China, Hefei, Anhui 230026, P. R. China

[†]These authors contributed equally to this work.

[*]Corresponding author. E-mail: lihl@ustc.edu.cn (H.L.); wxli70@ustc.edu.cn (W.L.); zengj@ustc.edu.cn (J.Z.).

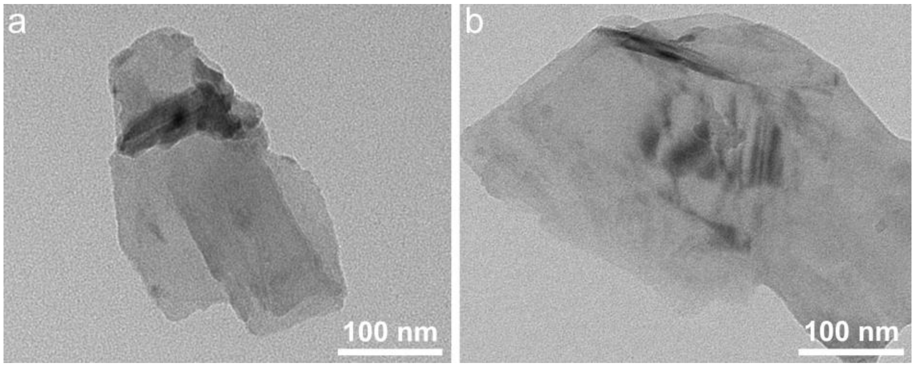

Supplementary Figure 1 | Morphology of BP and Au$_1$/BP nanosheets. (**a**) TEM image of BP nanosheets. (**b**) TEM image of Au$_1$/BP nanosheets.

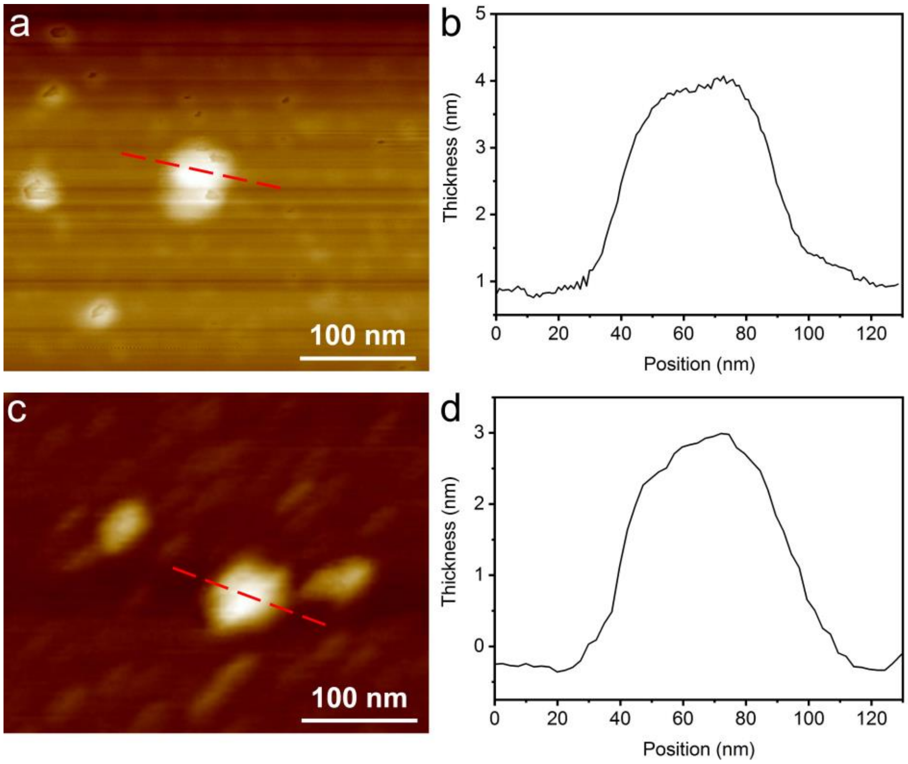

Supplementary Figure 2 | Morphology of BP and Au_1/BP nanosheets. (**a**) AFM image and (**b**) height profile of BP nanosheets. (**c**) AFM image and (**d**) height profile of Au_1/BP nanosheets.

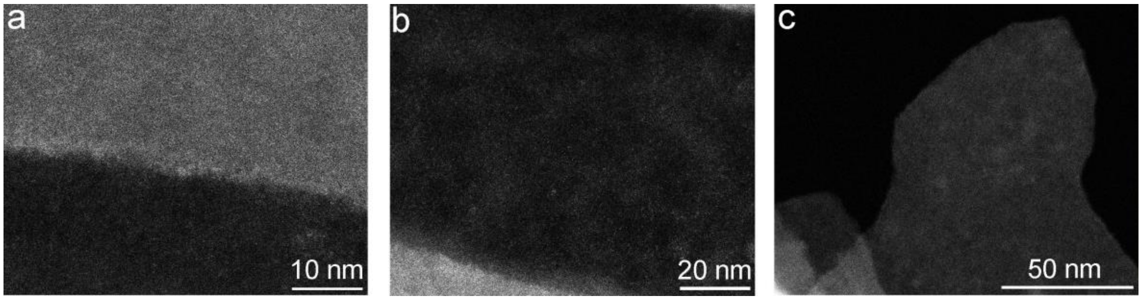

Supplementary Figure 3 | HAADF-STEM images of Au_1/BP nanosheets.

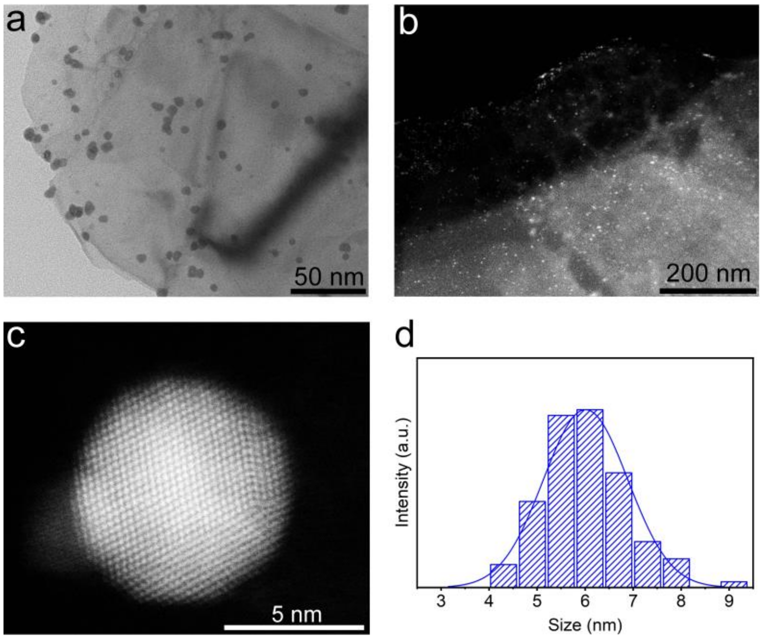

Supplementary Figure 4 | Characterizations of Au NPs/BP nanosheets. (**a**) TEM image of Au NPs/BP nanosheets. (**b**) HAADF-STEM image of Au NPs/BP nanosheets. (**c**) HAADF-STEM image of an individual Au nanoparticle. (**d**) Size distribution diagram of Au NPs/BP nanosheets.

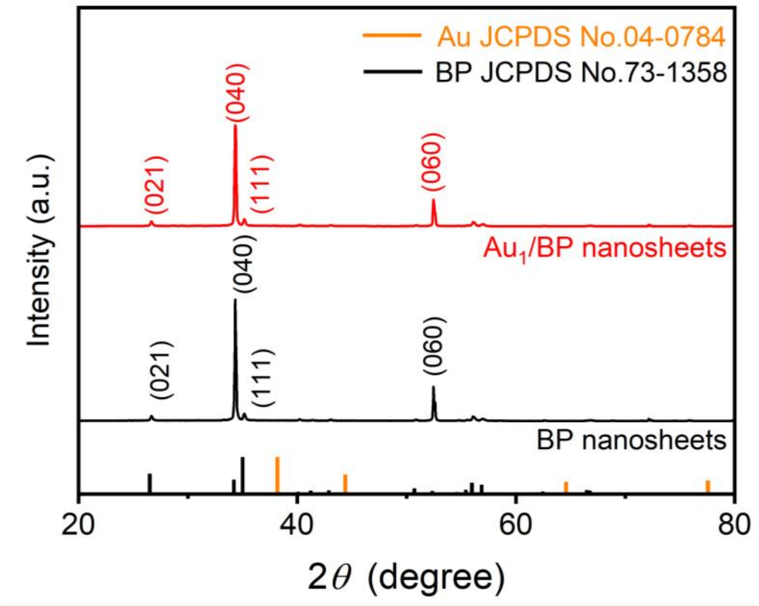

Supplementary Figure 5 | XRD patterns of BP and Au_1/BP nanosheets.

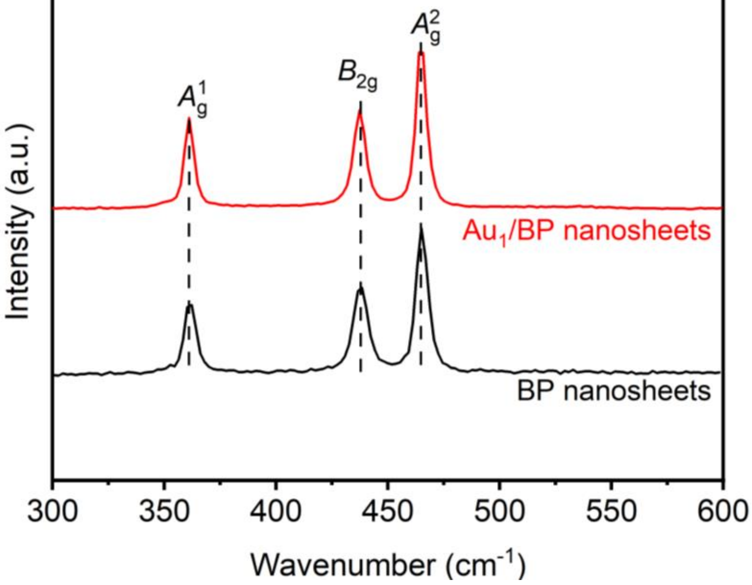

Supplementary Figure 6 | Raman spectra of BP and Au_1/BP nanosheets.

Supplementary Table 1 | EXAFS fitting results of Au_1/BP nanosheets before and after catalytic tests.

Sample	Au-P		Au-Au		*D. W.*	ΔE_0 (eV)
	R (Å)	*CN*	*R* (Å)	*CN*		
Au foil	–	–	2.86±0.01	12.0	0.008±0.001	4.0±0.5
Au_1/BP nanosheets	2.33±0.01	2.0±0.1	–	–	0.008 (Au)<br>0.003 (P)	6.8±1.7
Au_1/BP nanosheets after 10 *in-situ* cycles	2.44±0.02	2.6±0.3	–	–		17.4±2.8

R, distance between absorber and backscatter atoms; *CN*, coordination number; *D. W.*, Debye-Waller factor; ΔE_0, inner potential correction to account for the difference in the inner potential between the sample and the reference compound.

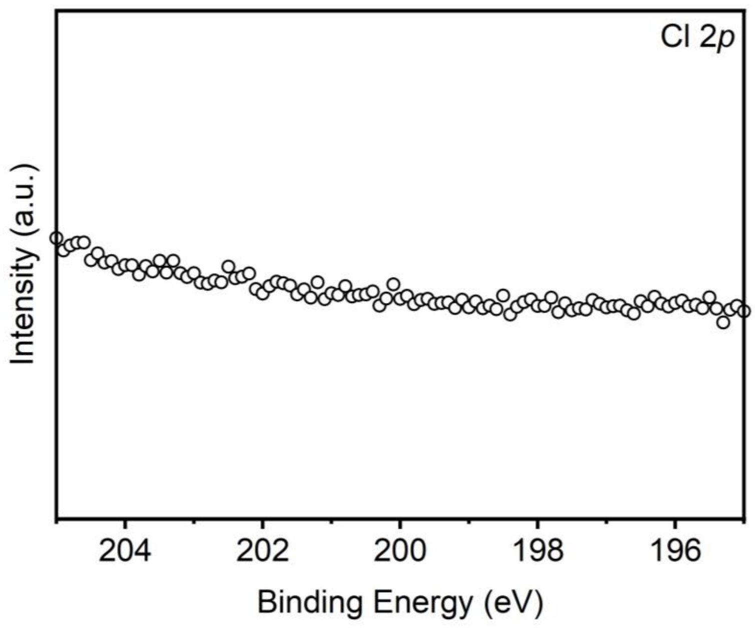

Supplementary Figure 7 | High-resolution Cl 2*p* XPS spectrum of Au_1/BP nanosheets.

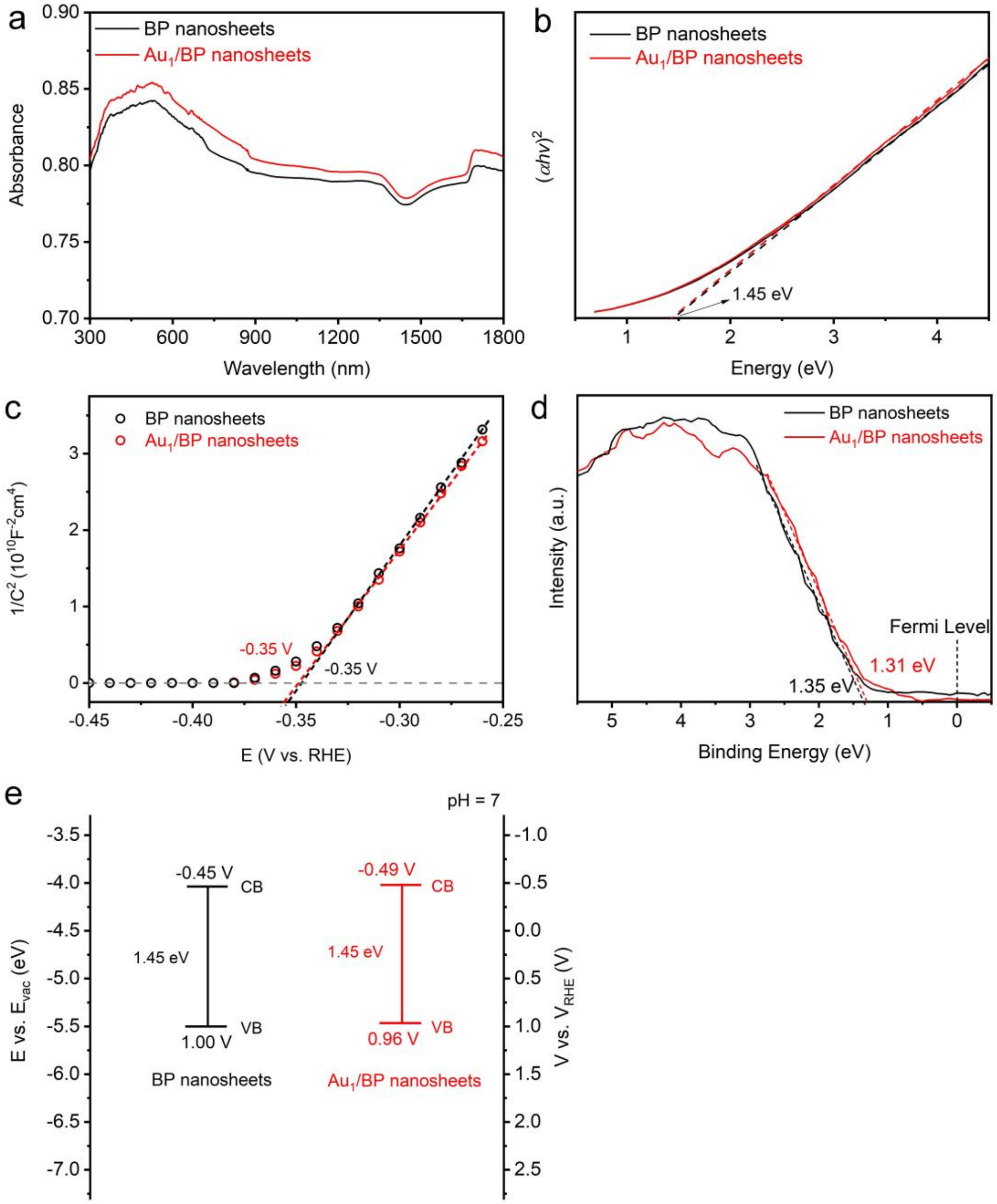

Supplementary Figure 8 | Band structures of BP and Au$_1$/BP nanosheets. (**a**) UV-vis-NIR absorption spectra and (**b**) the corresponding Tauc plots of BP and Au$_1$/BP nanosheets. (**c**) Mott-Schottky plots of BP and Au$_1$/BP nanosheets. (**d**) Valence XPS spectra of BP and Au$_1$/BP nanosheets. (**e**) Band structures of BP and Au$_1$/BP nanosheets.

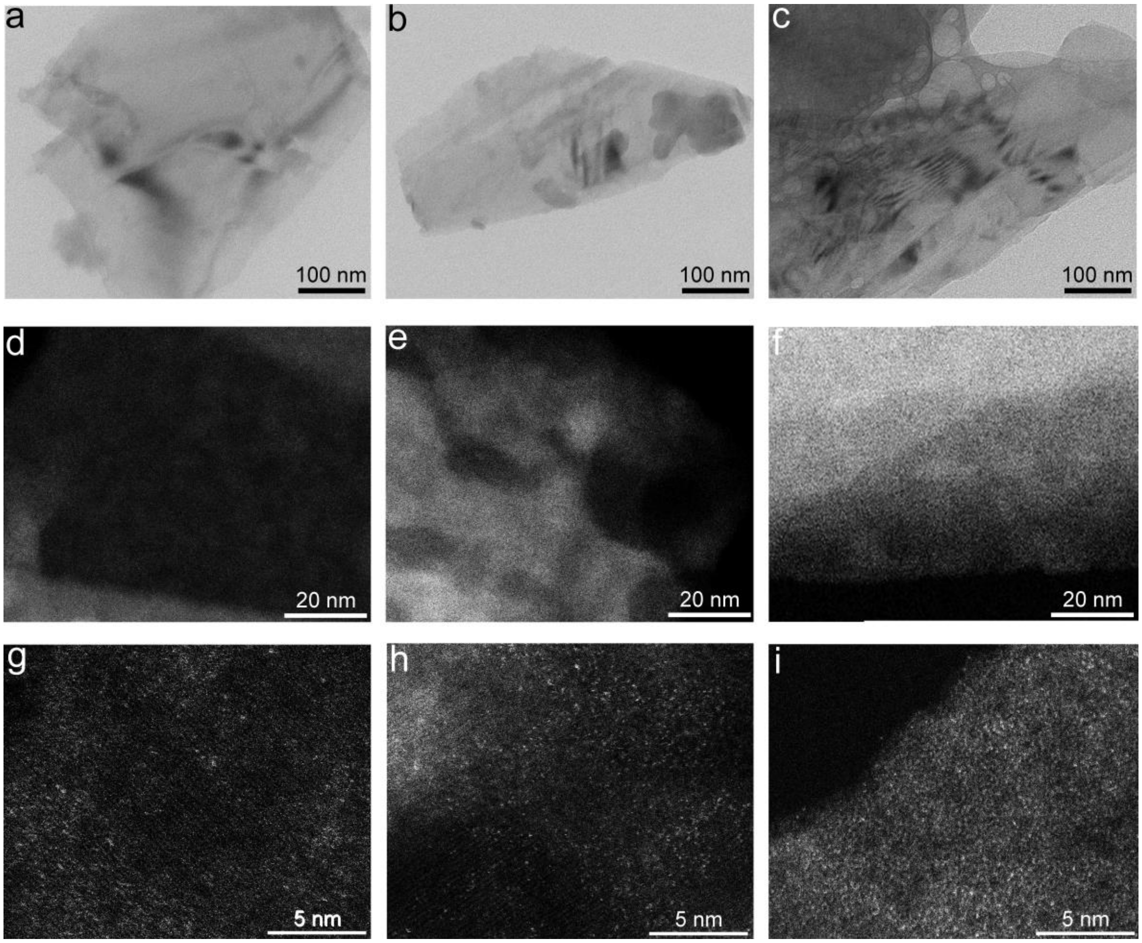

Supplementary Figure 9 | Characterizations of Pt_1/BP, Rh_1/BP, and Pd_1/BP nanosheets. TEM images of (**a**) Pt_1/BP, (**b**) Rh_1/BP, and (**c**) Pd_1/BP nanosheets. HAADF-STEM images of (**d**) Pt_1/BP, (**e**) Rh_1/BP, and (**f**) Pd_1/BP nanosheets. Magnified HAADF-STEM images of (**g**) Pt_1/BP, (**h**) Rh_1/BP, and (**i**) Pd_1/BP nanosheets.

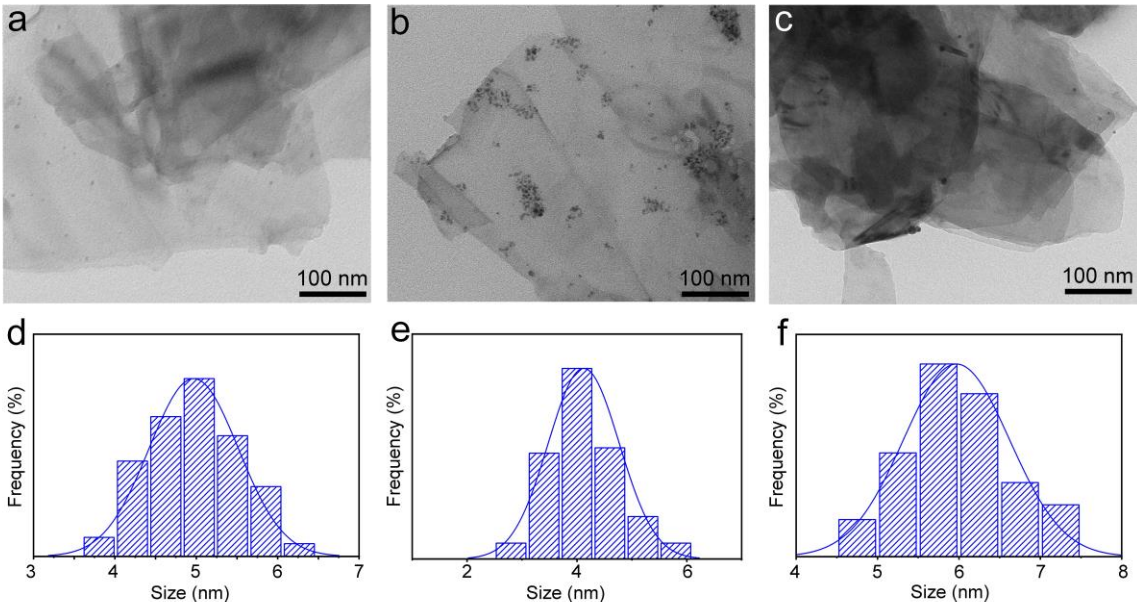

Supplementary Figure 10 | Characterizations of Pt NPs/BP, Rh NPs/BP, and Pd NPs/BP nanosheets. TEM images of (**a**) Pt NPs/BP, (**b**) Rh NPs/BP, and (**c**) Pd NPs/BP nanosheets. Particle size distribution diagrams of (**d**) Pt NPs/BP, (**e**) Rh NPs/BP, and (**f**) Pd NPs/BP nanosheets.

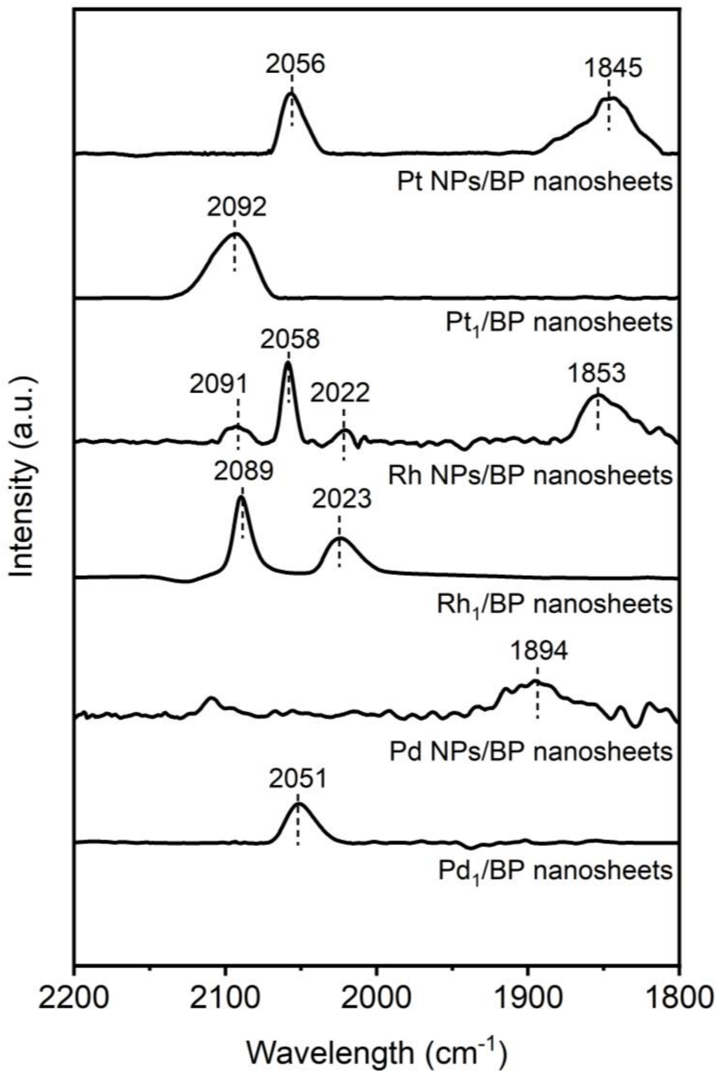

Supplementary Figure 11 | DRIFTS spectra of different catalysts using CO as a probe molecule.

Supplementary Table 2 | Assignment of DRIFTS peaks in Supplementary Figure 10.

Catalyst	Surface species	Wavenumber (cm^{-1})	Literature value (cm^{-1})	Reference
Pt NPs/BP	Linear Pt-(CO)	2056	2020	S1
	Bridge Pt-(CO)	1845	1850	
Pt_1/BP	Linear Pt-(CO)	2092	2088	
Rh NPs/BP	Rh-$(CO)_2$	2091, 2022	2116, 2049	S2
	Linear Rh-(CO)	2058	2082	
	Bridge Rh-(CO)	1853	1885	
Rh_1/BP	Rh-$(CO)_2$	2089, 2023	2116, 2049	
Pd NPs/BP	Bridge Pd-(CO)	1894	1978	S3
Pd_1/BP	Linear Pd-(CO)	2051	2030	S4

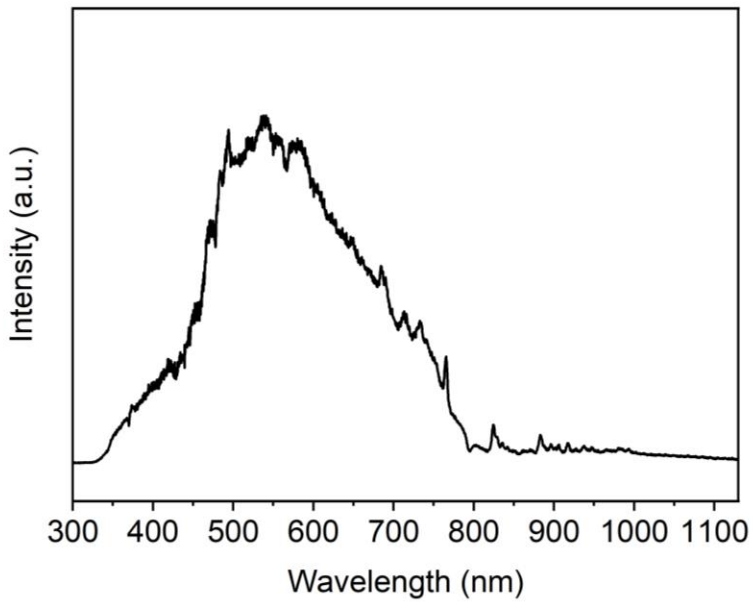

Supplementary Figure 12 | Spectrum of the incident light source.

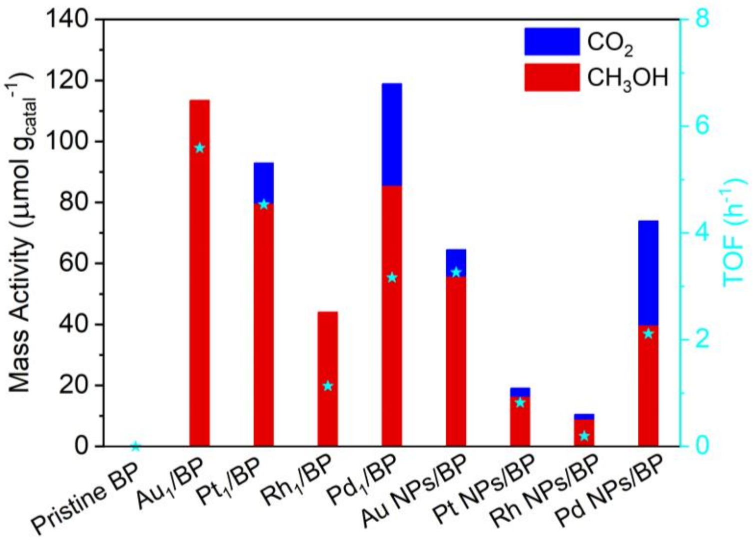

Supplementary Figure 13 | Comparison of the catalytic performance of different BP nanosheets based catalysts. Reaction conditions: 200 mg catalyst, 30 bar CH_4, 3.0 bar O_2, 20 mL water, 90 ℃, 2 h, light power 1.2 W, irradiation area 3.14 cm^{-2}.

Supplementary Table 3 | Comparison of catalytic performance reported in this work and representative results from published works.

Catalyst	Reaction conditions	Catalytic Activity	Methanol Selectivity	Reference
Au_1/BP	30 bar CH_4, 3 bar O_2 90 °C, Under irradiation	5.6 $mol_{methanol}/mol_{Au}/h$	>99%	This work
0.33_{metal}wt.% FeO_x/TiO_2	Atmospheric CH_4 H_2O_2 Room temperature Under irradiation	~5.95 $mol_{methanol}/mol_{Fe}/h$	＞90%	S5
0.10 wt%Rh/ZSM-5	50 bar CH_4, 10 bar CO, 8 bar O_2, 150 °C	2.71 $mol_{CH3OH}/mol_{Rh}/h$	-	S6
0.6 wt% Rh/TiO_2	20 bar CH_4, 5 bar CO 2 bar O_2, 150 °C	~1.31 $mol_{methanol}/mol_{Rh}/h$	100%	S2
AuPd/ZSM-5-C_{16}	3.0 MPa 3.3% H_2/6.6% O_2/1.6% CH_4/61.7% Ar/26.8% He, 70 °C	~13.9 $mol_{CH3OH}/mol_{AuPd}/h$	92%	S7
Au-Pd colloids	30 bar CH_4, 5 bar O_2, H_2O_2, 70 °C	2.303 $mol_{CH3OH}/mol_{metal}/h$	92%	S8
Cu-0.5/PCN	Atmospheric CH_4 No extra oxidant Room temperature Under irradiation	~0.31 $mol_{methanol}/mol_{Cu}/h$	-	S9
UiO-66(2.5TFA)-Fe	3 MPa CH_4, H_2O_2, 50 °C	~0.65 $mol_{CH3OH}/mol_{Fe}/h$	~5.4%	S10
$Au_{0.75}$/ZnO	15 bar CH_4, 5 bar O_2 30 °C Under irradiation	~18.0 $mol_{CH3OH}/mol_{Au}/h$	99.1%	S11
FeN_4/GN-2.7	2 MPa CH_4 H_2O_2, 25 °C	~0.47 $mol_{methanol}/mol_{Fe}/h$	-	S12
0.13 wt % AuPd/Rutile TiO_2 (800 °C treated)	30.5 bar CH_4 H_2O_2, 70 °C	6.8 $mol_{CH3OH}/mol_{metal}/h$	9.3%	S13
0.3 wt% Rh/ZrO_2	28.5 bar CH_4 H_2O_2, 70 ℃	~1.25 $mol_{CH3OH}/mol_{Rh}/h$	64.1%	S14
$BiVO_4$-V_2O_5	Atmospheric CH_4 No extra oxidant ~70 °C Under irradiation	3.3 $umol_{CH3OH}/g_{cat}/h$	100%	S15

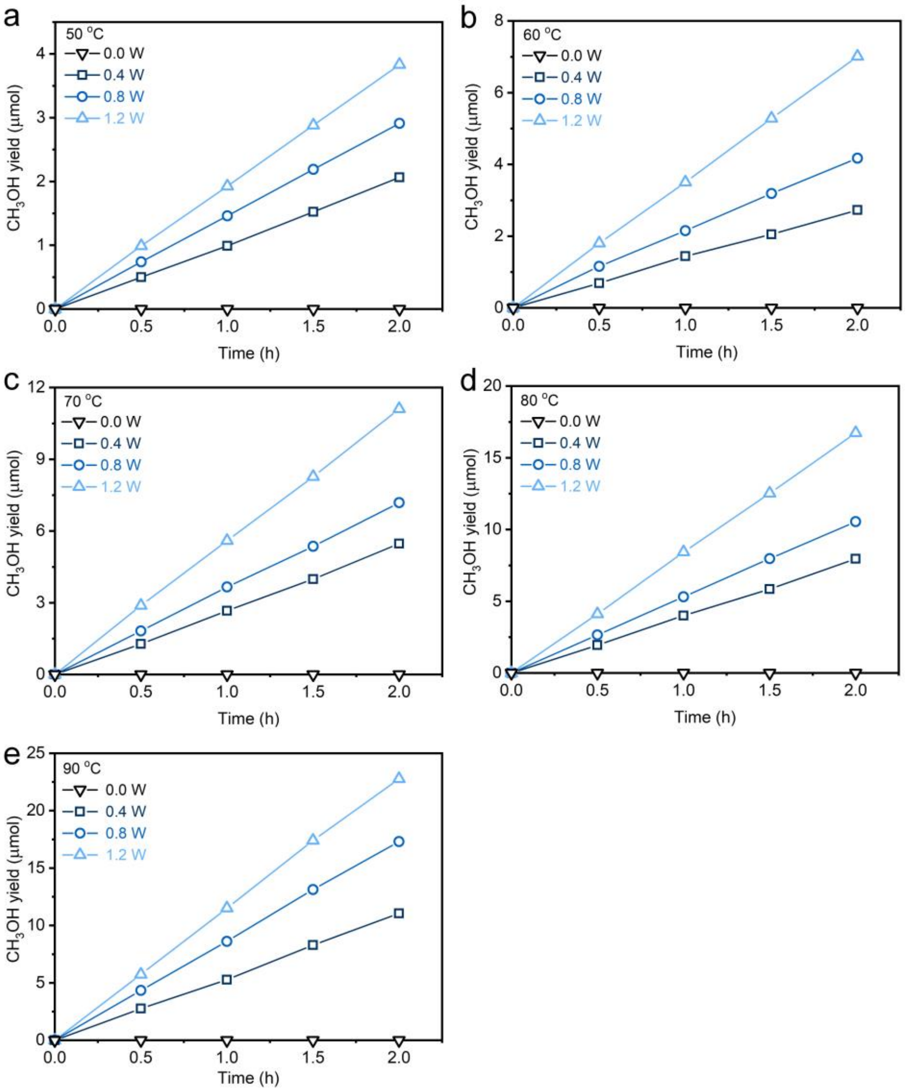

Supplementary Figure 14 | Time courses for partial oxidation of methane over Au_1/BP nanosheets at (a) 50 ºC, (b) 60 ºC, (c) 70 ºC, (d) 80 ºC, and (e) 90 ºC. Reaction conditions: 200 mg catalyst, 30 bar CH_4, 3 bar O_2, full spectrum light, irradiation area 3.14 cm^{-2}.

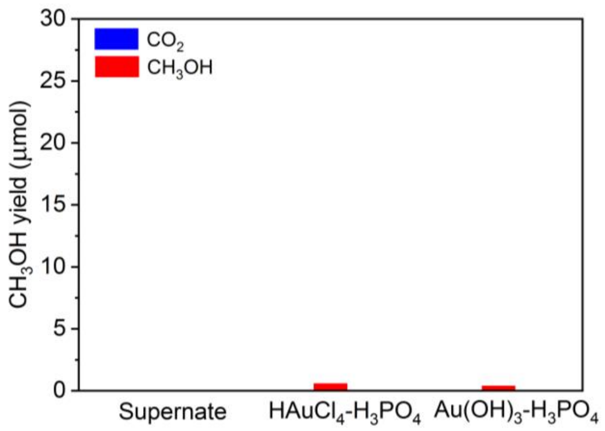

Supplementary Figure 15 | Catalytic performance of supernate after one reaction cycle, $HAuCl_4$-H_3PO_4, and $Au(OH)_3$-H_3PO_4 aqueous solution. After a 2-h reaction over Au_1/BP nanonsheets, the catalysts were removed by centrifugation. After the centrifugation, we collected the supernate as the catalyst for an additional 2-h reaction under the same conditions. The $HAuCl_4$-H_3PO_4 and $Au(OH)_3$-H_3PO_4 experiments were conducted with a total amount of Au of 0.4 mg and P of 200 mg.

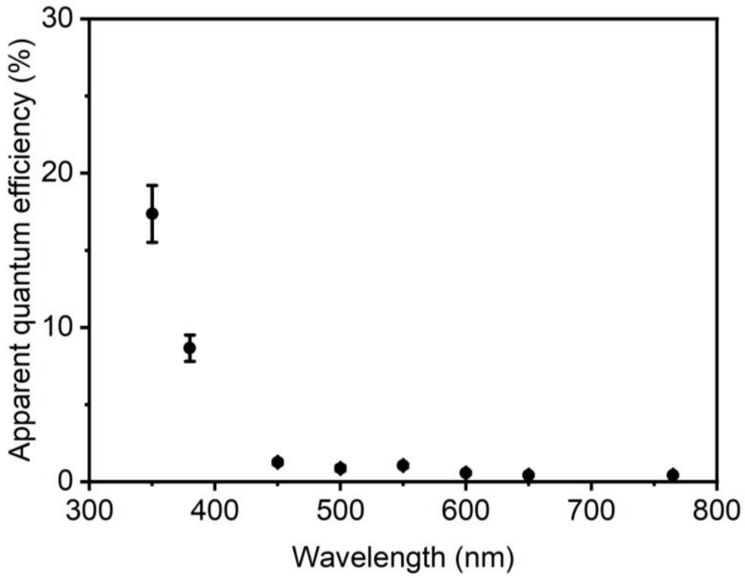

Supplementary Figure 16 | Wavelength-dependent apparent quantum yields (AQY) of methanol for Au_1/BP nanosheets under the irradiation of various monochromatic lights. Error bars represent standard deviation from three independent measurements.

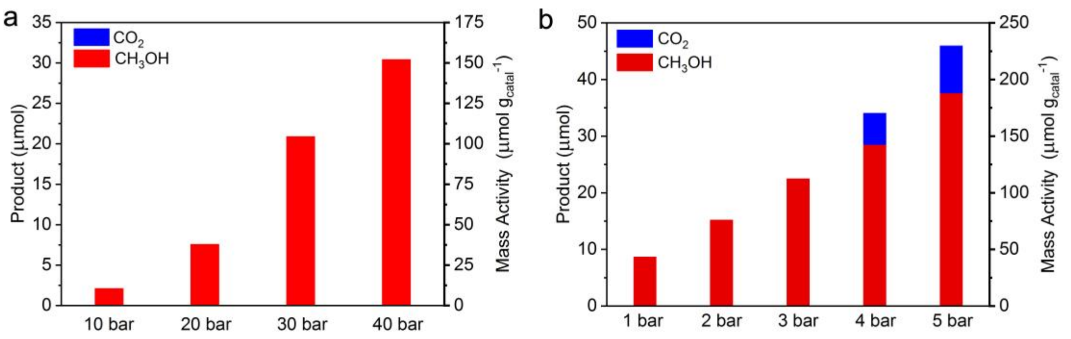

Supplementary Figure 17 | Influence of CH_4 and O_2 partial pressure. (a) Product yields over Au_1/BP nanosheets under different CH_4 pressures. Reaction conditions: 200 mg catalyst, 20 mL water, 3 bar O_2, 90 ℃, light power 1.2 W, irradiation area 3.14 cm^{-2}, 2 h. (b) Product yields over Au_1/BP nanosheets under different O_2 pressures. Reaction conditions: 200 mg catalyst, 20 mL water, 30 bar CH_4, 90 ℃, light power 1.2 W, irradiation area 3.14 cm^{-2}, 2 h.

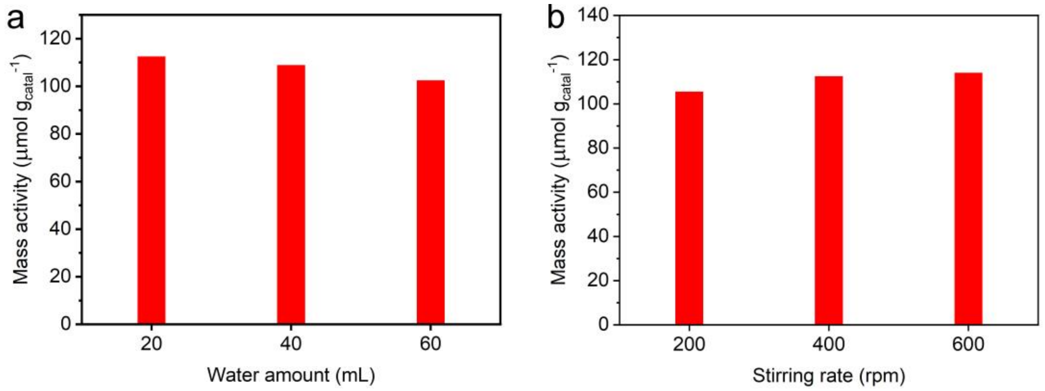

Supplementary Figure 18 | Influence of water amount and stirring rate. (a) Product yields over Au_1/BP nanosheets under different water amount. Reaction conditions: 200 mg catalyst, 30 bar CH_4, 3 bar O_2, 90 ℃, full spectrum light, light power 1.2 W, irradiation area 3.14 cm^{-2}, 2 h. (b) Product yields over Au_1/BP nanosheets under different stirring rate. Reaction conditions: 200 mg catalyst, 20 mL H_2O, 30 bar CH_4, 3 bar O_2, 90 ℃, full spectrum light, light power 1.2 W, irradiation area 3.14 cm^{-2}, 2 h.

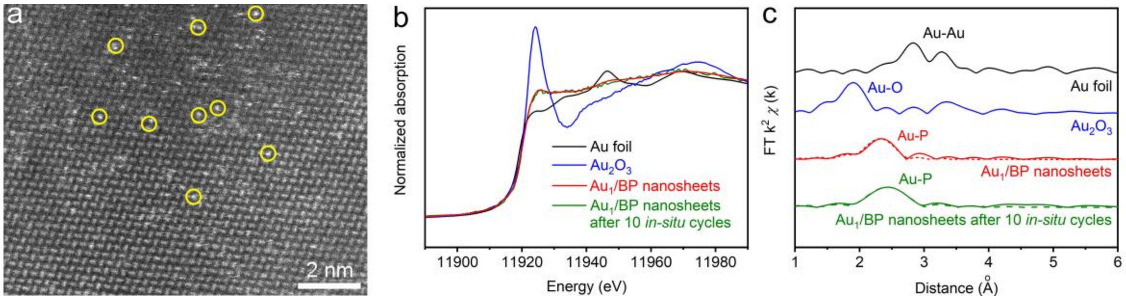

Supplementary Figure 19 | Characterization of Au_1/BP nanosheets after 10 *in-situ* cycles in comparison with the fresh catalyst. (**a**) HAADF-STEM image of Au_1/BP nanosheets after 10 *in-situ* cycles. (**b**) XANES spectrum and (**c**) EXAFS spectrum of Au_1/BP nanosheets after 10 *in-situ* cycles. Au foil, Au_2O_3, and fresh Au_1/BP nanosheets were used as the references. For each cycle, the catalytic reaction was operated under 33 bar (CH_4:O_2 = 10:1) at 90 ^oC for 2 h.

Supplementary Table 4 | Assignment of *in-situ* DRIFTS peaks observed in this study.

Surface species	Calculated wavenumber (cm^{-1})	Experimental wavenumber (cm^{-1})	Literature value (cm^{-1})	Reference
P-O-P	820	911	891	S16
P=O	1183	1246	1286	
P-OH	3561, 3267	3350	3734	S17
CH_4*	-	3015, 1304	3015, 1304	S9
CH_3*	1408	1456	1457	S8

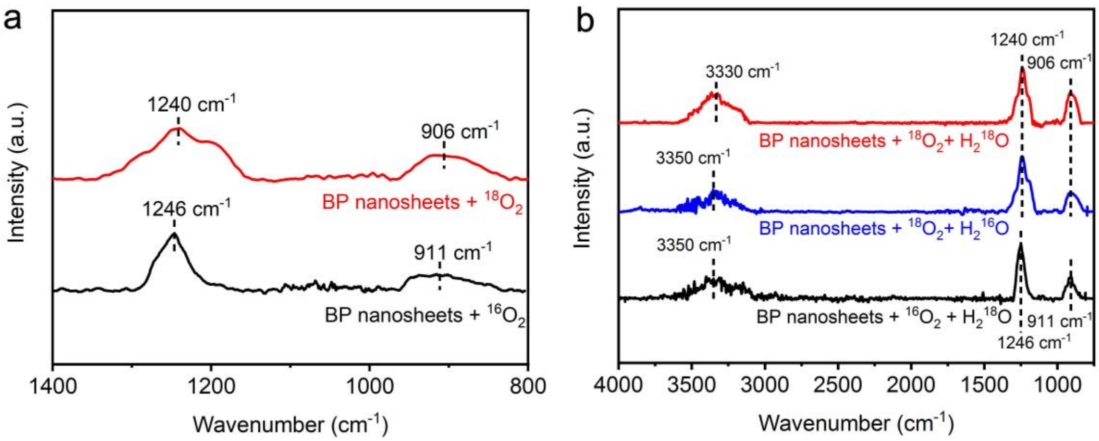

Supplementary Figure 20 | Isotope labeling DRIFTS spectra. (a) *In-situ* DRIFTS spectra of BP nanosheets purged by 1 bar of $^{16}O_2$ or $^{18}O_2$ at 90 ^oC under light irradiation. (b) *In-situ* DRIFTS spectra of BP nanosheets purged by 1 bar of $^{16}O_2/H_2{}^{18}O$, $^{18}O_2/H_2{}^{16}O$, or $^{18}O_2/H_2{}^{18}O$ at 90 ^oC under light irradiation. Background spectra were acquired after flowing under 1 bar of N_2 with the rate of 20 sccm at 150 ^oC for 0.5 h, followed by cooling to 90 ^oC.

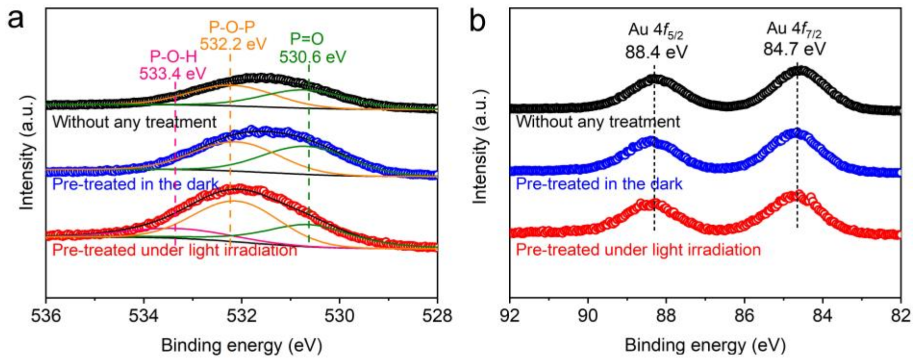

Supplementary Figure 21 | *Quasi-situ* XPS studies of Au_1/BP nanosheets. *Quasi-situ* XPS spectra of (**a**) O 1*s* and (**b**) Au 4*f* for Au_1/BP nanosheets after the treatment with O_2/H_2O in the dark or under light irradiation (1.2 W) at 90 ^{o}C in comparison with those without any treatment.

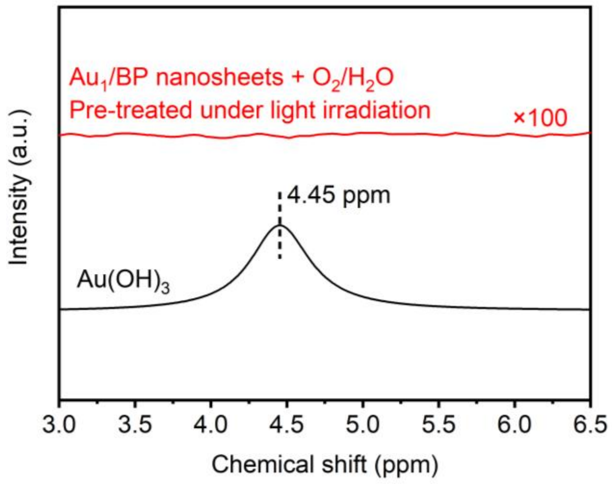

Supplementary Figure 22 | ^{1}H solid NMR spectrum of Au_1/BP nanosheets pre-treated with O_2/H_2O under light irradiation (1.2 W) at 90 ºC. $Au(OH)_3$ was used as the references.

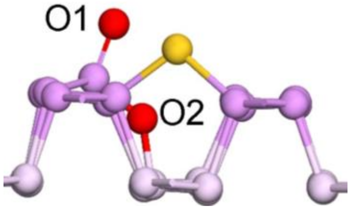

Supplementary Figure 23 | Adsorption configuration of two O atoms on Au_1/BP nanosheets. Yellow, violet, pink, and red spheres represent Au, surface P, subsurface P, and O atoms, respectively.

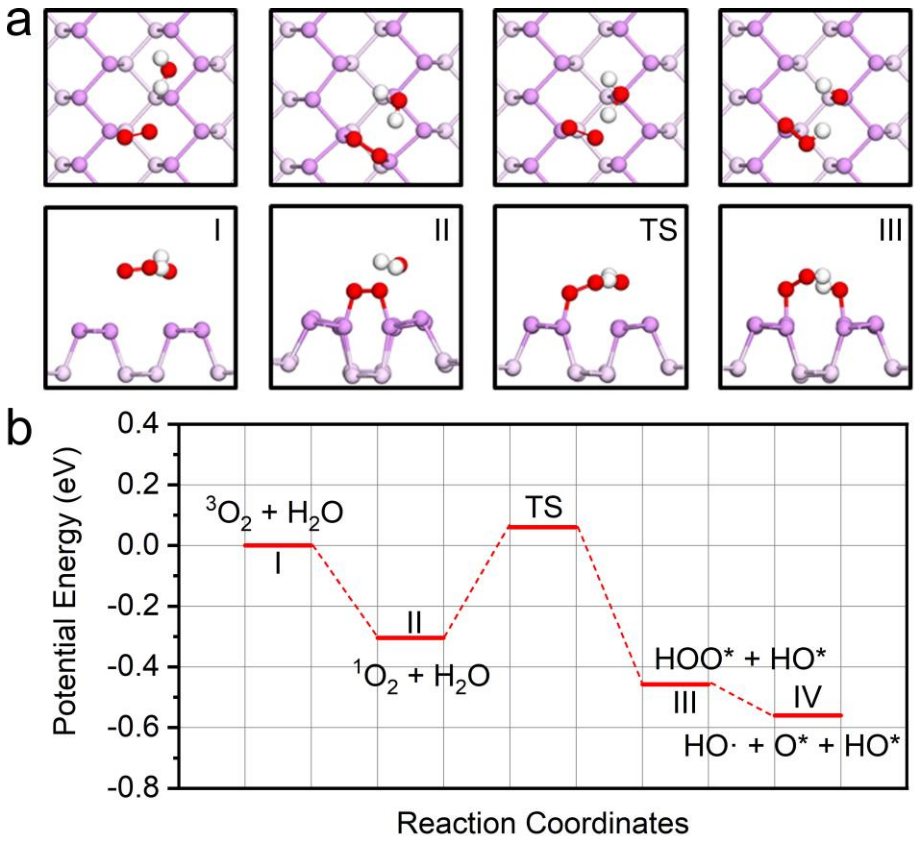

Supplementary Figure 24 | (a) Configurations and (b) reaction path of oxygen activation under light irradiation. Violet, pink, red, and white spheres represent Au, surface P, subsurface P, O, and H atoms, respectively.

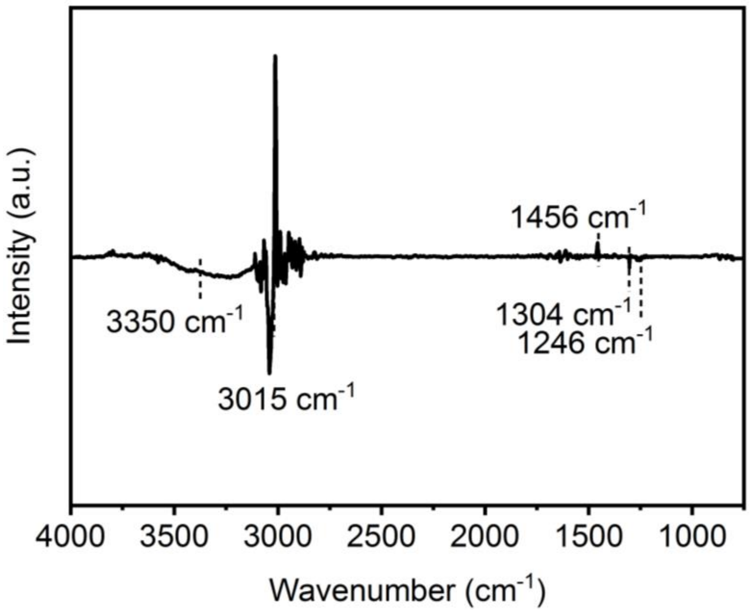

Supplementary Figure 25 | *In-situ* DRIFTS spectra of pre-treated Au_1/BP nanosheets purged by 1 bar of CH_4 at 90 ºC in the dark. Pre-treatment: Catalysts were purged by 1 bar of O_2/H_2O at 90 ºC for 0.5 h under light irradiation, followed by 1 bar of N_2 at 150 ºC for 0.5 h. Afterwards, the catalysts were purged by 1 bar of CH_4 at 25 ºC for 0.5 h to obtain background spectra.

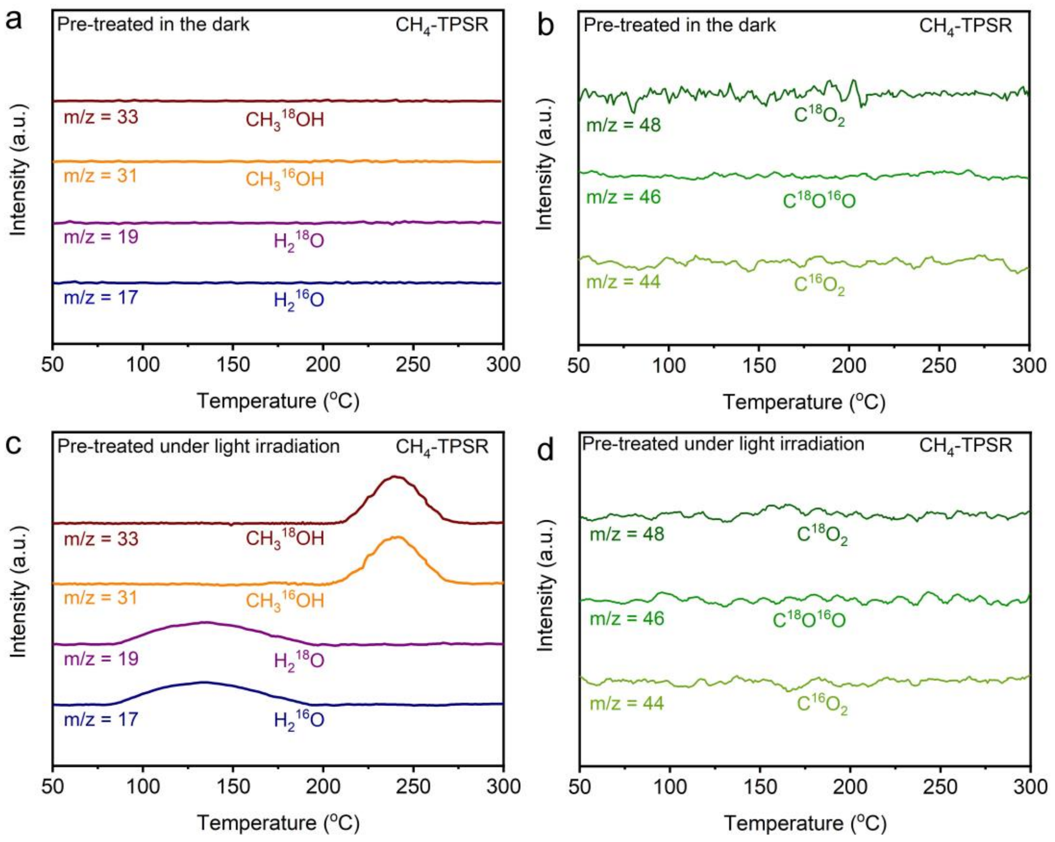

Supplementary Figure 26 | CH_4-TPSR studies of Au_1/BP nanosheets. TPSR-MS profiles of Au_1/BP nanosheets after the pre-treatment (**a, b**) in the dark and (**c, d**) under light irradiation. For pre-treatment, the catalyst was dispersed in $H_2^{18}O$ within a vial under O_2 at 90 °C for 0.5 h, followed by being dried in vacuum oven.

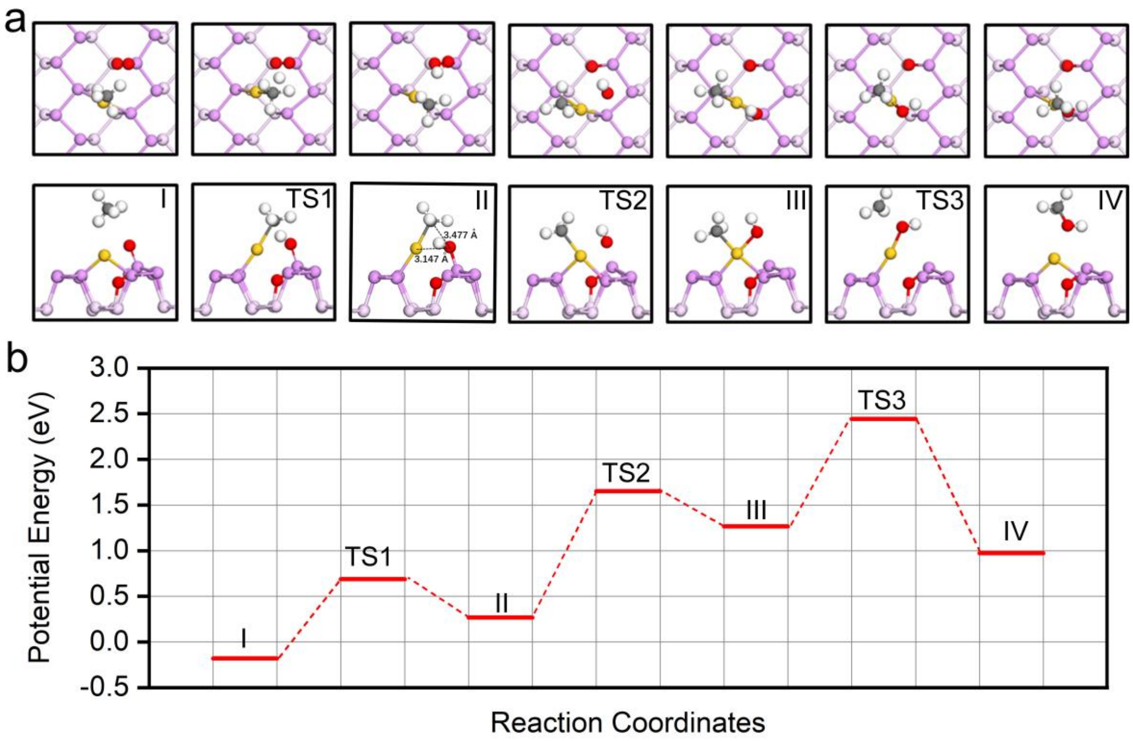

Supplementary Figure 27 | (a) Configurations and (b) reaction path for CH_4 oxidation in the dark after O_2 activation in the dark. For each configuration, the top image shows the top view, while the bottom image shows the side view. Yellow, violet, pink, red, grey, and white spheres represent Au, surface P, subsurface P, O, C, and H atoms, respectively. In configuration II, the distance between the O atom in P-OH and the C atom in CH_3* is 3.477 Å, while that between the O atom in P-OH and the Au atom is 3.147 Å.

Supplementary Table 5 | Reaction energy of CH_4 activation over all possible active sites on Au/BP nanosheets.

Reaction	Reaction Energy
CH_4 + 2P → P-CH_3 + P-H	2.08
CH_4 + 2P=O → P-O-CH_3 + P-OH	2.28
CH_4 + P=O + P → P-O-CH_3 + P-H	2.59
CH_4 + P=O + P → P-CH_3 + P-OH	1.78
CH_4 + P-OH → P-CH_3 + H_2O	0.31
CH_4 + P-OH → CH_3OH + HP	1.26
CH_4 + P=O → CH_3OH + + P	0.96
CH_4 + P=O + P-OH → P-O-CH_3 + H_2O + P	0.82
CH_4 + Au + P → Au-CH_3 + P-H	0.62
CH_4 + Au + P → P-CH_3 + Au-H	0.34
CH_4 + Au + P=O → Au-CH_3 + P-OH	0.32
CH_4 + Au + P=O → P-O-CH_3 + Au-H	0.84
CH_4 + Au + P-OH → Au-H + CH_3OH	-0.48
CH_4 + Au + P-OH → Au-CH_3 + H_2O	-1.14

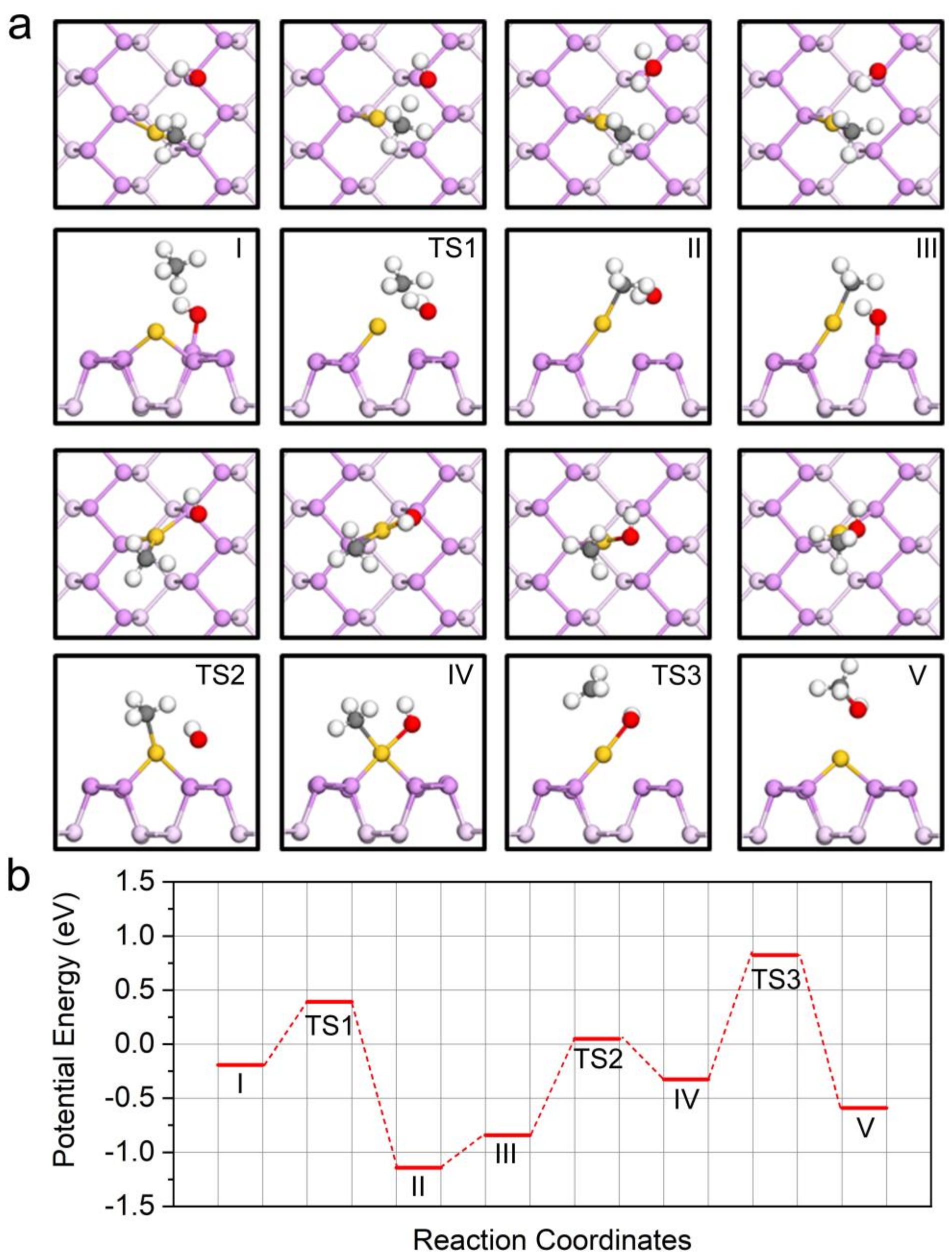

Supplementary Figure 28 | (a) Configurations and (b) reaction path for CH_4 oxidation in the dark after O_2 activation under light irradiation. For each configuration, the top image shows the top view, while the bottom image shows the side view. Yellow, violet, pink, red, grey, and white spheres represent Au, surface P, subsurface P, O, C, and H atoms, respectively.

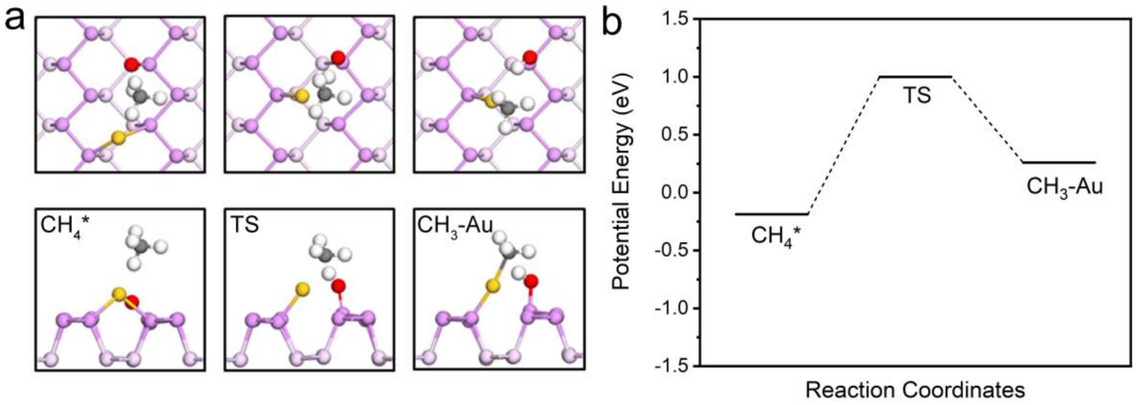

Supplementary Figure 29 | (a) Configurations and (b) reaction path for partial oxidation of methane by P=O species over Au_1/BP nanosheets. For each configuration, the top image shows the top view, while the bottom image shows the side view. Yellow, violet, pink, red, grey, and white spheres represent Au, surface P, subsurface P, O, C, and H atoms, respectively.

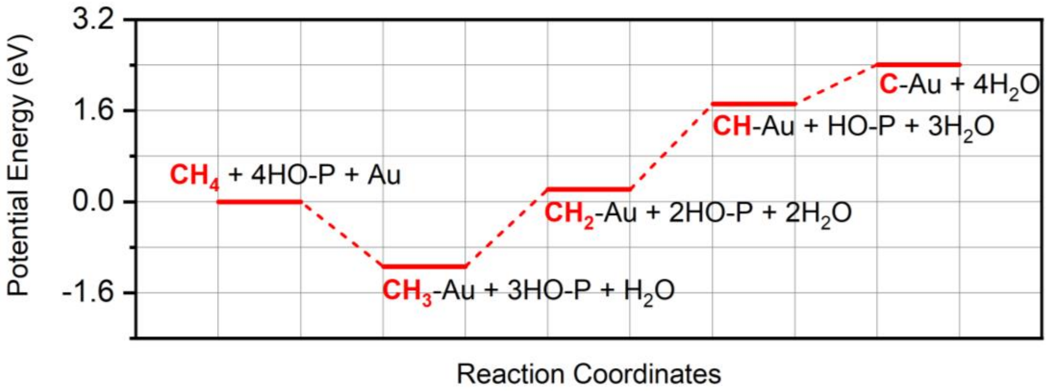

Supplementary Figure 30 | Reaction energy of methane dehydrogenation over Au_1/BP nanosheets.

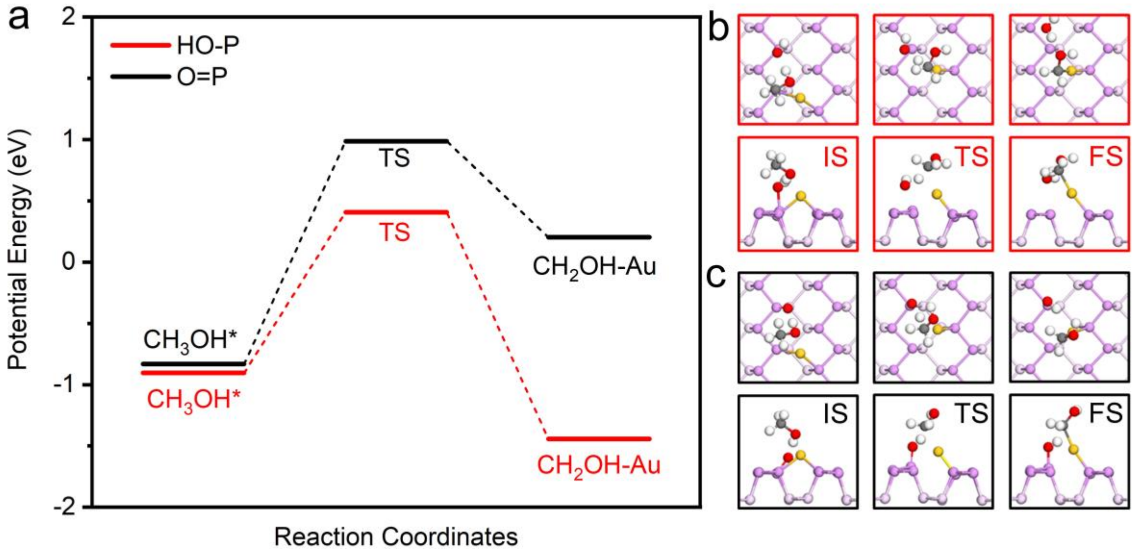

Supplementary Figure 31 | Calculated energy barriers for methanol oxidation. (**a**) Reaction path of methanol oxidation by P-OH and P=O species. (**b**) Configurations of intermediates during methanol oxidation by P-OH species. (**b**) Configurations of intermediates during methanol oxidation by P=O species. For each configuration, the top image shows the top view, while the bottom image shows the side view. Yellow, violet, pink, red, grey, and white spheres represent Au, surface P, subsurface P, O, C, and H atoms, respectively.

Supplementary References

S1. Zhang, X. *et al.* Platinum-copper single atom alloy catalysts with high performance towards glycerol hydrogenolysis. *Nature Commun.* **10**, 5812 (2019).

S2. Shan, J., Li, M., Allard, L.F., Lee, S. & Flytzani-Stephanopoulos, M. Mild oxidation of methane to methanol or acetic acid on supported isolated rhodium catalysts. *Nature* **551**, 605-608 (2017).

S3. Jiang, L. *et al.* Facet engineering accelerates spillover hydrogenation on highly diluted metal nanocatalysts. *Nature Nanotechnol.* **15**, 848-853 (2020).

S4. Manrique, R. *et al.* The nature of the active sites of Pd-Ga catalysts in the hydrogenation of CO_2 to methanol. *Catal. Sci. Technol.* **10**, 6644-6658 (2020).

S5. Xie, J. *et al.* Highly selective oxidation of methane to methanol at ambient conditions by titanium dioxidesupported iron species. *Nature Catal.* **1**, 889-896 (2018).

S6. Tang, Y. *et al.* Single rhodium atoms anchored in micropores for efficient transformation of methane under mild conditions. *Nature Commun.* **9**, 1231 (2018).

S7. Jin, Z. *et al.* Hydrophobic zeolite modification for in situ peroxide formation in methane oxidation to methanol. *Science* **367**, 193-197 (2020).

S8. Sushikevich, V. L., Palagin, D., Ranocchiari, M. & Bokhoven, J. A. Selective anaerobic oxidation of methane enables direct synthesis of methanol. *Science* **358**, 223-227 (2017).

S9. Zhou, Y., Zhang, L. & Wang, W. Direct functionalization of methane into ethanol over copper modified polymeric carbon nitride via photocatalysis. *Nature Commun.* **10**, 506 (2019).

S10. Zhao, W. *et al.* Fe-O clusters anchored on nodes of metal-organic frameworks for direct methane oxidation. *Angew. Chem. Int. Ed.* DOI: 10.1002/anie.202013807.

S11. Zhou, W. *et al.* Highly selective aerobic oxidation of methane to methanol over gold decorated zinc oxide via photocatalysis. *J. Mater. Chem. A* **8**, 13277-13284 (2020).

S12. Cui, X. *et al.* Room-temperature methane conversion by graphene-confined single iron atoms. *Chem* **4**, 1902-1910 (2018).

S13. Williams, C., *et al.* Selective oxidation of methane to methanol using supported AuPd catalysts prepared by stabilizer-free sol-immobilization. *ACS Catal.* **8**, 2567-2576 (2018).

S14. Kwon, Y., Kim, T. Y., Kwon, G., Yi, J., & Lee, H. et al. Selective activation of methane on single-atom catalyst of rhodium dispersed on zirconia for direct conversion. *J. Am. Chem. Soc.* **139**, 17694-17699 (2017).

S15.Murcia-Lopez, S., Villa, K., Andreu, T. & Morante, J. R. Partial oxidation of methane to methanol using bismuth-based photocatalysts. *ACS Catal.* **7**, 2878-2885 (2017).

S16.Rao, K. S., Reddy, M. S., Kumar, V. R. & Veeraiah, N. Dielectric, magnetic and spectroscopic properties of Li_2O-WO_3-P_2O_5 glass system with Ag_2O as additive. *Mater. Chem. Phys.* **111**, 283-292 (2008).

S17.Diallo-Garcia, S., Osman, B. M., Krafft, J.-M., Boujday, S, Guylène. C. Discrimination of infrared fingerprints of bulk and surface POH and OH of hydroxyapatites. *Catal. Today* **226**, 81-88 (2014).
